# Identification and Seasonal Abundance of Web- and Air-Borne Sex Pheromone Components of Western Black Widow Spiders, *Latrodectus hesperus*

**DOI:** 10.1007/s10886-025-01590-6

**Published:** 2025-03-12

**Authors:** Andreas Fischer, Alexandra J. Fischer, Regine Gries, Emmanuel Hung, Kelvin Lau, Aryan Monfared, Gerhard Gries

**Affiliations:** 1 Department of Biological Sciences, Simon Fraser University, Burnaby, BC Canada; 2 Department of General and Systematic Zoology, University of Greifswald, Greifswald, Germany

**Keywords:** Courtship pheromone, Mate-attractant pheromone components, Seasonality of pheromone production, Strategic signalling, Cobweb spider

## Abstract

Sex pheromones mediate mate location in many animal taxa. Widow spider males are attracted to sex pheromones disseminating from female webs. Upon arrival on a web, males court in response to web-borne contact pheromone components. *N*-3-Methylbutanoyl-*O*-methylpropanoyl-L-serine methyl ester (**1**) is the single known, weakly effective, contact pheromone component of female western black widows, *Latrodectus hesperus.* Moreover, the seasonal periodicity of pheromone signalling by female spiders is unknown. We tested the hypotheses that females of *L. hesperus* (1) deposit multiple contact pheromone components on their web that transition to mate-attractant pheromone components, and (2) increase pheromone signalling during the primary mating season. Analyses of web extract by gas and liquid chromatography-mass spectrometry (GC-MS; LC-MS) revealed *N*-3-methylbutanoyl-*O*-methylpropanoyl-L-serine (**7**), the corresponding acid of **1**. Web extract of unmated female *L. hesperus*, and the synthetic blend of **1** and **7**, were equally effective in eliciting courtship by males but web extract induced more sustained courtship. Tested singly, **7** prompted longer courtship behaviour by males than **1**. Synthetic isobutyric acid (**10**), the hydrolysis product of **1** and **7**, attracted male spiders in a field experiment. The abundance of **1** and **7** on female webs, with expected corresponding dissemination of **10** from webs, peaked during the summer when males are most abundant, indicating strategic sexual signalling by female spiders.

**Supplementary Information:**

The online version contains supplementary material available at 10.1007/s10886-025-01590-6.

## Introduction

Pheromonal communication has been intensely studied in insects (Francke and Schulz 2010; Buchinger and Li 2023) but remains comparatively unexplored in spiders (Huber 2005; Schulz 2013; Fischer 2019). Although female spiders are known to produce sex pheromones that attract males and induce courtship (Huber 2005; Gaskett 2007; Uhl and Elias 2011), pheromones have been identified in only 15 (out of 50,000) spider species (Fischer 2019; Fischer et al. 2023b). Moreover, the amount of pheromone deposited has been quantified only in recent studies (Weiss and Schneider 2022, 2023; Fischer et al. 2023a, 2024). Females of the wasp spider, *Argiope bruennichi*, gradually increase the pheromone titre on their web until they have attracted a mate (Weiss and Schneider 2022, 2023). Whereas many spiders die after a brief mating season, others are long-lived and multivoltine, and mate repeatedly (Foelix 2015). For example, mated female redback spiders, *Latrodectus hasselti*, wait for two months before calling again (Perampaladas et al. 2008). However, it remains unclear whether pheromone production changes with the (mating) seasons or steadily increases with the age of long-lived unmated females (Kokko 1997).

**Fig. 1:**
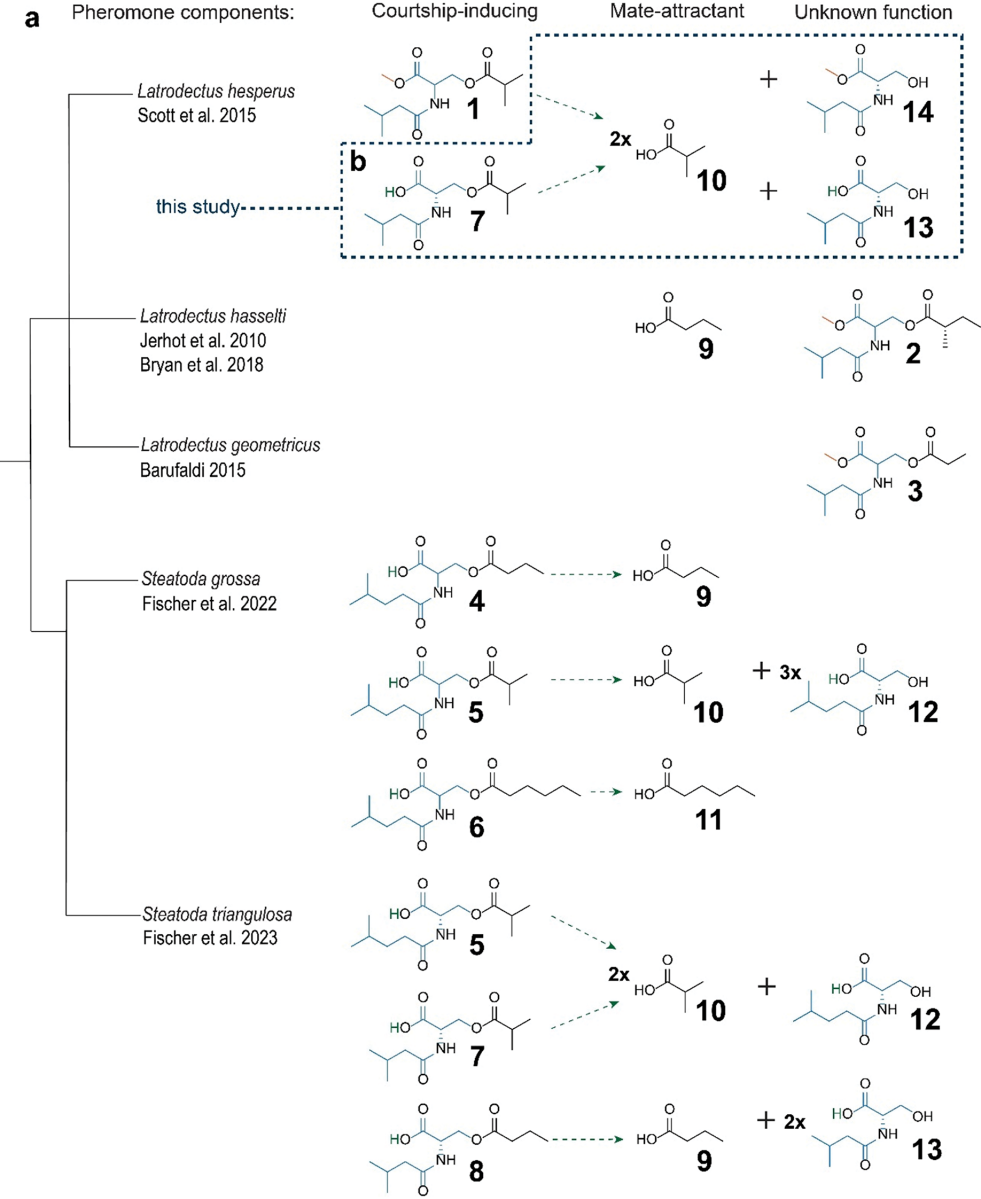
Pheromone components and hydrolysis products of female widow spiders. (**a**) Previously reported pheromone components of *Latrodectus hesperus* (Scott et al. 2015b), *L*. *hasselti* (Jerhot et al. 2010; Bryan et al. 2018), *L*. *geometricus* (Baruffaldi 2016), *Steatoda grossa* (Fischer et al. 2022) and *S*. *triangulosa* (Fischer et al. 2023b): **1** (*N*-3-methylbutanoyl-*O*-methylpropanoyl-L-serine methyl ester), **2** (*N*-3-methylbutanoyl-*O*-(*S*)-2-methylbutanoyl-L-serine methyl ester), **3** (*N*-3-methylbutanoyl-*O*-isobutanoyl-L-serine methyl ester), **4** (*N*-4-methylvaleroyl-*O*-butanoyl-L-serine), **5** (*N*-4-methylvaleroyl-*O*-isobutanoyl-L-serine), **6** (*N*-4-methylvaleroyl-*O*-hexanoyl-L-serine), **7** (*N*-3-methylbutanoyl-*O*-methylpropanoyl-L-serine), **8** (*N*-3-methylbutanoyl-*O*-butanoyl-L-serine), **9** (butyric acid), **10** (isobutyric acid), **11** (hexanoic acid), **12** (*N*-4-methylvaleroyl-L-serine), and **13** (*N*-3-methylbutanoyl-serine). The contact pheromone components of *S. grossa* (**4–6**) and *S. triangulosa* (**5**, **7**, **8**) hydrolyse at their ester bonds, giving rise to the mate-attractant pheromone components **9–11** (*S. grossa*) and **9–10** (*S. triangulosa*), with **12** and **13** accumulating as hydrolysis products on webs. (**b**) Dashed box enclosing pheromone components and hydrolysis products of *L. hesperus* identified in this study: the contact pheromone components **7** and **1** hydrolyse at the ester bond, giving rise to the mate-attractant pheromone component **10**, whereas **13** and *N*-3-methylbutanoyl-serine methyl ester (**14**) accumulate on webs

Widow spiders (Araneae: Theridiidae, Latrodectinae) have become a model phylum for spider chemical ecology research, with five out of the 15 known spider pheromones identified in this phylum (Fig. 1a) (Fischer et al. 2023b). The western black widow, *Latrodectus hesperus*, is the most intensely studied species in this group (Kasumovic and Andrade 2004; Blackledge and Zevenbergen 2007; Salomon et al. 2010; Johnson et al. 2011; Salomon 2011; Scott et al. 2012, 2015a, b, 2019, 2020; Baruffaldi and Andrade 2015, 2020, 2024; DiRienzo et al. 2019; Clark and Johnson 2024). In our study population (see below), polyandrous female *L. hesperus* live through multiple (summer) mating seasons (Salomon et al. 2010), whereas polygynous males generally die after a single season (Baruffaldi and Andrade 2020). Males become sexually mature and seek mates primarily during the summer but some mature males are also present during winter (Salomon et al. 2010). Mate-seeking males locate webs of conspecific females guided by airborne pheromone components emanating from the females’ web (Kasumovic and Andrade 2004). Upon arrival on a web and sensing web-borne contact pheromone components, males engage in courtship (Scott et al. 2015b). A male is courting a female by cutting sections of her web and bundling them with his own silk, concurrently engaging in vibratory signalling. These vibratory signals enable the female to discern between a prospective mate and prey (Vibert et al. 2014). The web-bundling behavior also suppresses the female’s aggression and reduces the latency to copulation (DiRienzo et al. 2019). An aphrodisiac pheromone is assumed to be present on the courtship silk of males (Scott et al. 2018). Interestingly, the male’s web-bundling behaviour also renders the female’s web less attractive to rival males (Scott et al. 2015a), as shown in other spiders (Watson 1986; Schulz and Toft 1993).

The single known contact pheromone component of female *L. hesperus* is *N*-3-methylbutanoyl-*O*-methylpropanoyl-L-serine methyl ester (**1**) (Fig. 1) (Scott et al. 2015b). Ester **1** structurally resembles pheromone components of *L. hasselti* [*N-*3-methylbutanoyl-*O*-(*S*)-2-methylbutanoyl-L-serine methyl ester (**2**)] (Jerhot et al. 2010) and of *L. geometricus* (brown widows) [*N*-3-methylbutanoyl-*O*-isobutanoyl-L-serine methyl ester (**3**)] (Baruffaldi 2016) (Fig. 1a); however, synthetic **1** elicits courtship in far fewer *L. hesperus* males (3%) than web extracts of female spiders (92%) (Scott et al. 2015b). This differential effect on male courtship implies the presence of additional unknown contact pheromone components in web extracts. Ester **1** was identified by gas chromatography-mass spectrometry (GC-MS) of web extract, a method that may miss polar pheromone components which chromatograph poorly. The very polar pheromone components of female *Steatoda grossa* (false widows) [*N*-4-methylvaleroyl-*O*-butanoyl-L-serine (**4**), *N*-4-methylvaleroyl-*O*-isobutanoyl-L-serine (**5**), *N*-4-methylvaleroyl-*O*-hexanoyl-L-serine (**6**)] (Fischer et al. 2022), and of female *S. triangulosa* (triangulate cobweb spiders) [**5**, *N*-3-methylbutanoyl-*O*-methylpropanoyl-L-serine (**7**), *N*-3-methylbutanoyl-*O*-butanoyl-L-serine (**8**)] (Fischer et al. 2023b) (Fig. 1a) were identified through parallel analytical techniques: high-performance liquid chromatography-tandem mass spectrometry (HPLC-MS/MS) and GC-MS analyses of BSTFA-derivatized web extracts (Fischer et al. 2022, 2023b). The synthetic ternary pheromone blends of *S. grossa* and *S. triangulosa* elicit courtship by males equivalent to web extracts (Fischer et al. 2022, 2023b).

The mate-attracting pheromone component of *L. hasselti* is butyric acid (**9**) (Bryan et al. 2018) but analogous components of *L. hesperus* and *L. geometricus* remain unknown. The contact pheromone components **4**, **5** and **6** of *S. grossa*, and **5**, **7** and **8** of *S. triangulosa*, all hydrolyse at the ester bond, releasing short chain carboxylic acids as mate-attractant pheromone components, with the serine residues accumulating on webs (Fischer et al. 2022, 2023b). Specifically, the mate-attractant pheromone components of *S. grossa* are butyric acid (**9**), isobutyric acid (**10**), and hexanoic acid (**11**), whereas *N*-4-methylvaleroyl-L-serine (**12**) accumulates on webs (Fischer et al. 2022). In *S. grossa*, compound **12** is not a pheromone precursor and is absent from the pheromone gland that contains only contact pheromone components which give rise to the mate-attractant pheromone components **9**, **10** and **11**, and to compound **12** (Fischer et al. 2022). Similarly, the mate-attractant pheromone components of *S. triangulosa* are **9** and **10**, whereas **12** and *N*-3-methylbutanoyl-serine (**13**) accumulate on webs (Fischer et al. 2023b) (Fig. 1a). Ester hydrolyses are likely mediated by a carboxyl-ester-hydrolase enzyme present on webs of both *S. grossa* and *L. hesperus*. The enzyme was found both in the major ampullate silk gland of *L. hesperus* females and on their silk fibers (Chaw et al. 2015). The hydrolysis rate is positively correlated with the pH of the spider’s silk, ranging from pH 4 to 7 (Fischer et al. 2022). If we were to accept the premise that spider females are able to modify their web’s pH, it follows that they can adjust the dissemination rate of mate-attractant pheromone. Spider females may modify their silk’s pH during the ‘acid bath’ of silk fiber production (Vollrath et al. 1998; Heim et al. 2009) or via phosphate pH buffers (Schildknecht et al. 1972). Although the mechanisms underlying release rate adjustments of mate-attractant pheromone are not fully understood, evidence is mounting that females are capable of these adjustments (see discussion).

Here, we re-analysed the contact (courtship-inducing) and the mate-attractant sex pheromone components of female *L. hesperus* and studied the seasonality of sexual communication. In laboratory and field experiments, we tested the hypotheses (H) that female *L. hesperus* (H1a, b) deposit multiple contact pheromone components on their webs that hydrolyse to mate-attractant pheromone components, and (H2) increase pheromone signalling during the primary mating season in the summer.

## Methods

### Spider Collection and Maintenance

Juvenile *L. hesperus* were collected from their webs under driftwood on Centennial Beach in Tsawwassen, British Columbia, Canada (49° 0’ 53.856’’ N, 123° 2’ 26.6532’’ W) between 2018 and 2022. Irrespective of developmental stage and sex, spiders were kept singly in Petri dishes in the insectary of Simon Fraser University maintained at 22 ºC and a reversed 12 L:12D light cycle. Weekly, all spiders were provisioned with black blow flies, *Phormia regina*, and were provided with a fresh moistened cotton ball. Subadult males were moved to a separate room with similar conditions to avoid exposure of males to female pheromones (Cory and Schneider 2017). Under these laboratory conditions, spiders became sexually mature within weeks and were invariably unmated.

### Collection and Extraction of Spider Webs

Webs that unmated subadult and adult female spiders had built for three days on triangular prisms (18 × 18 × 18 × 25 cm) of bamboo skewers (Bradshaw International Inc., CA, USA) (Fischer et al. 2018) were separately reeled up on glass rods and extracted 24 h in 100 µL of acetonitrile (99% HPLC grade, Fisher Chemical, ON, Canada).

### Chemical Analyses of Web Extracts

In our search for pheromone components, we focused on mass homologues of acylated serine derivative pheromone components previously identified in spiders (Fig. 1). To this end, web extracts of 10 adult unmated female *L. hesperus* (expected to contain pheromone) and of 10 subadult female *L. hesperus* (expected not to contain pheromone) were analysed by high performance liquid chromatography-mass spectrometry (HPLC-MS). For these analyses, we used a Bruker maXis Impact Quadrupole Time-of-Flight LC/MS system, consisting of an Agilent 1200 LC fitted with a Spursil C_18_ column (30 mm × 3.0 mm, 3 μm; Dikma Technologies, Foothill Ranch, CA, USA) and a Bruker maXis Impact Ultra-High Resolution tandem TOF (UHR-Qq-TOF) mass spectrometer. The LC/MS was operated with positive electrospray ionization (+ ESI) at a gas temperature of 200 ºC and a flow of 9 L/min. The nebuliser was set to 4 bar and the capillary voltage to 4200 V. The column was eluted with a 0.4-mL/min flow of a solvent gradient, starting with 80% water and 20% acetonitrile, and ending with 100% acetonitrile after 4 min. The solvent system contained 0.1% formic acid to improve the peak shape of compounds.

Web extracts of adult unmated females were also analysed by gas chromatography-MS, using an Agilent 7890B GC fitted with a DB-5 GC-MS column (30 m × 0.25 mm ID, film thickness 0.25 μm) and coupled to a 5977 A MSD. The injector port of the GC was set to 250 ºC, the transfer line to 280 °C, the MS source to 230 ºC, and the MS quadrupole to 150 ºC. Helium served as the carrier gas at a flow rate of 35 cm^− 1^. The following temperature program was used: 50 ºC (held for 5 min), then increased 10 ºC min^− 1^ to 280 ºC (held for 10 min). Not to miss polar compounds, such as carboxylic acids which chromatograph poorly, we also treated web extracts with BSTFA (bis-trimethylsilyl-trifluoroacetamide) (Stalling et al. 1968), thereby converting polar compounds to corresponding silyl-ethers or esters which chromatograph well. Total ion chromatograms of BSTFA-derivatized web extracts, and the mass spectra of compounds in these extracts, are presented in Supplementary Material. Compounds were identified by comparing their mass spectra and retention indices with those of authentic standards that were either available from previous studies (Jerhot et al. 2010; Scott et al. 2015b; Fischer et al. 2022, 2023b), or were synthesized following established protocols for the syntheses of acylated serine derivates (Jerhot et al. 2010; Scott et al. 2015b; Fischer et al. 2022, 2023b).

### Experimental Design for Testing Contact Pheromone Components

Courtship behaviour of male spiders in response to contact pheromone components was tested on a bamboo T-rod apparatus (Scott et al. 2015b; Fischer et al. 2018) in a bioassay room illuminated by red light. The apparatus consisted of a vertical beam (30 × 0.4 cm) and a horizontal beam (25 × 0.4 cm), with a piece of filter paper (2 cm^2^) attached to each distal end of the horizontal beam. The vertical beam was inserted into plasticine in the centre of a water-filled tray to prevent the bioassay spider from leaving the apparatus. The treatment stimulus (web extract of unmated females or synthetic compounds in 100 µL of solvent) and the control stimulus (100 µL of solvent) were applied to the pieces of filter paper. This T-rod apparatus has been proven effective to test for courtship behaviour by male widow spiders. Sensing contact pheromone, the male essentially behaves as if he were courting on the web of a female. In response to the presence of female-produced or synthetic pheromone on a filter paper, the male engages in courtship, pulling silk with his hindlegs from his spinnerets and adding it to the filter paper (Scott et al. 2015b; Fischer et al. 2022, 2023b). The design of the T-rod apparatus is, however, not conducive for testing attraction of males to airborne sex-attractant pheromone components emanating from filter paper, likely because the still-air diffusion of these components toward the T-rod junction is not sufficiently fast, resulting in indiscriminate (50:50) responses by males towards treatment and control stimuli (Fischer et al. 2018, 2019, 2020a, b, 2022, Fischer et al. 2023b).

For each 15-min bioassay, a single male spider was placed on the vertical beam of the T-rod apparatus and allowed to move freely on the apparatus. We recorded whether males courted (Exps. 1, 2), and the duration of their courtship (Exps. 1**–**6), in response to test stimuli, with the observer ‘blind’ to the test stimuli. On all bioassay days, the same number of replicates was run for parallel experiments 1 and 2, and parallel experiments 3**–**6. Within experiments, treatment and control sites were alternated between replicates. Each male was assayed only once in each experiment, and the T-rod, and piece of filter paper were discarded after each replicate. Bioassay males were less than 21 days post sexual maturity and were offered food weekly.

Experiment 1 (*n* = 20; summer 2019) tested courtship of males in response to web extract of adult unmated female spiders (1 web-equivalent/replicate) *versus* a solvent control. Parallel experiment 2 (*n* = 20) tested synthetic candidate pheromone components **1** (25 ng) and **7** (527 ng) – identified and quantified in web extracts (see Results) – at 1 web equivalent *versus* a solvent control. Parallel experiments 3–6 (*n* = 20 each; summer 2022) tested **1**, **7** and **13** in ternary combination [Exp. 3: **1** (47 ng), **7** (888 ng), **13** (65 ng); total = 1 µg] and singly (Exps. 4–6: **1**, **7**, **13** at 1 µg each). Component **14** was not assayed in 2022 because it was found, and quantified, only one year later (in 2023).

For each of experiments 1 and 2, 20 male spiders were tested. Following the collapse of the spider field population in late 2021 due to a severe cold snap (see below), only 20 male spiders were available for testing in experiments 3–6. Each of these 20 males had previously been exposed to web extract in another study.

### Field Testing of the Candidate Mate-Attractant Pheromone Component Isobutyric Acid

**Fig. 2:**
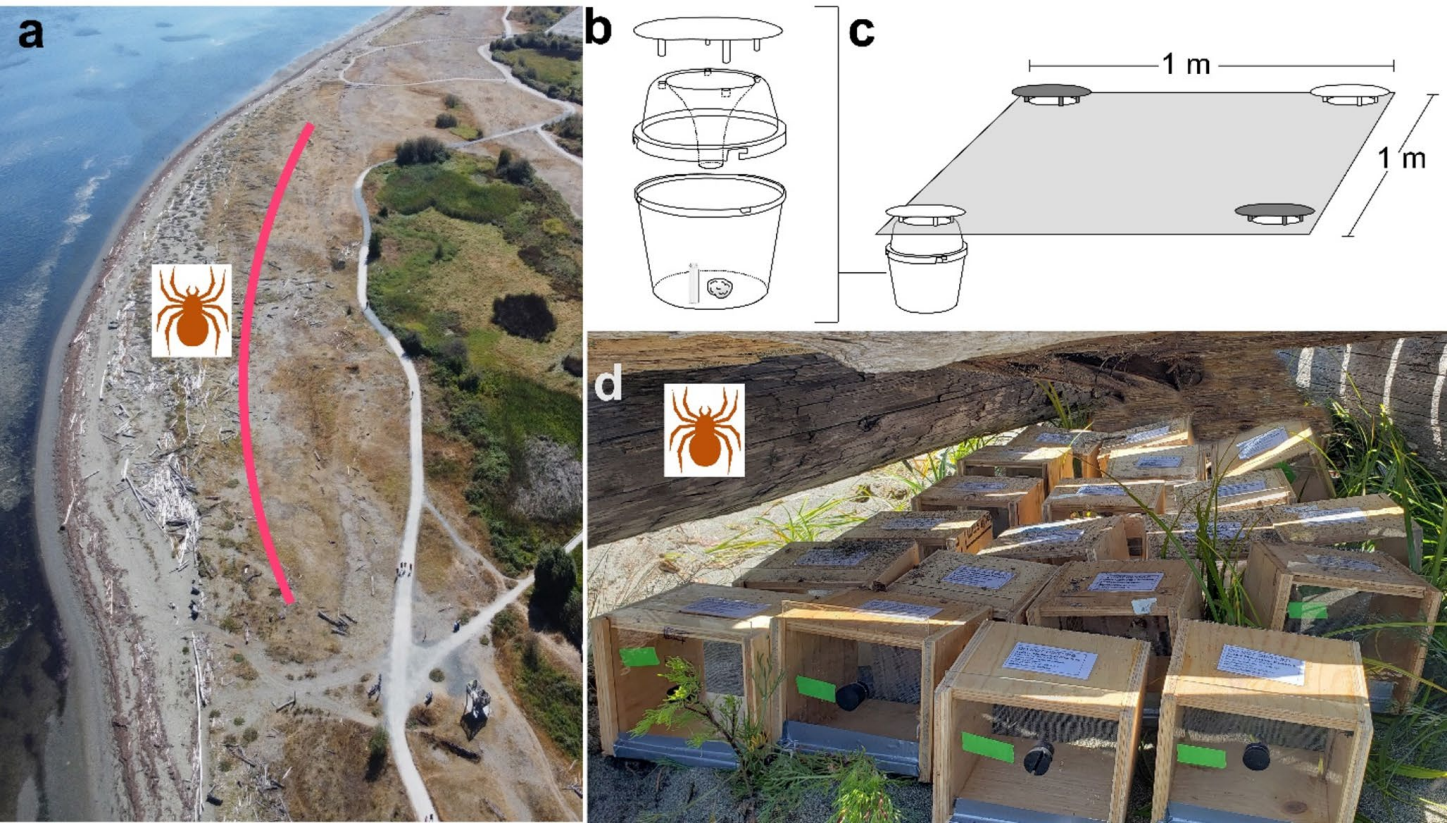
Location and experimental design of field experiments. (**a**) Bird’s-eye view of the field site at Centennial Beach, Tsawwassen, British Columbia, Canada. The red line marks the 500-m distance along which Unitraps were deployed; the spider symbol denotes the location where spiders were housed to quantify the pheromone on their webs every month between January and December 2021. (**b**) Unitrap baited with the synthetic mate-attractant pheromone component isobutyric acid and fitted with artificial spider web to retain responding spiders. (**c**) In each of eight experimental replicates, four Unitraps were arranged in a 1 × 1 m square such that the trap opening was flush with the soil surface; both traps in opposite corners of the square were baited or not (control). (**d**) Twenty wooden boxes sheltered under driftwood, each box housing a single unmated female *Latrodectus hesperus*

We field-tested attraction of male *L. hesperus* to synthetic isobutyric acid (**10**) – the candidate mate-attractant pheromone component – on Centennial Beach in Tsawwassen (Exp. 7, Fig. 2a). The experiment was run during the night of 24 September 2018, during the *L. hesperus* mating season (Salomon et al. 2010). We would have preferred to run the experiment repeatedly during the peak mating season of *L. hesperus* but we were constrained by poor weather and the late issuance of the Centennial Beach Park permit (#2018-BOU-6) which also limited field testing because the field site was deemed environmentally sensitive habitat.

Isobutyric acid was hypothesized to originate from the hydrolysis of contact pheromone components **1** and **7** (Fig. 1), drawing on analogous findings in two closely related spiders species (Fischer et al. 2022, 2023b), and on reports that the enzyme thought to hydrolyse **1** and **7** is present on *L. hesperus* webs (Chaw et al. 2015). As only the carboxylic acid hydrolysis products – but not the courtship-inducing contact pheromone components – attracted *S. grossa* and *S. triangulosa* males in previous studies (Fischer et al. 2022, 2023b), contact pheromone components **1** and **7** were not tested in this field experiment.

Eight experimental replicates were set up in 70-m intervals along a 500-m transect, 20 m inland from the high-tide line (Fig. 2a). Each replicate consisted of four Unitraps (Fig. 2b; Forestry Distributing, Boulder, CO, USA) positioned in the corners of a 1 × 1 m square (Fig. 2c), with two treatment traps, and two control traps, in the opposite corners of the square. All traps were placed in the ground such that the top of the trap bucket was flush with the soil surface, and the trap lid was 3 cm above ground (Fig. 2c). Each trap was fitted with polyester Halloween-decoration spider web (0.2 g, no brand name) known to retain spiders (Fischer et al. 2019). Treatment traps were baited with isobutyric acid (840 ng) formulated in 600 µL of mineral oil (Anachemia, Montreal, CA) in 2-dram glass vials, whereas control traps were fitted with 2-dram glass vials containing only mineral oil (600 µL).

### Monthly Quantifications of Contact Pheromone Components on Webs in 2021

To track the amount of contact sex pheromone components female *L. hesperus* deposit on their webs over the course of an entire year, a field experiment (Exp. 8) was run in 2021. To this end, 20 subadult females were collected on Centennial Beach in the fall of 2020 and reared to unmated adults in the insectary (see above). In late December 2020, each of 20 adult female spiders was placed into a separate plywood box (15 × 15 × 15 cm) with a removable plexiglass lid (Fig. 2d). All spiders were cold-adapted by placing boxes outdoors for several hours per day for seven days, gradually expanding the cold-exposure period to 24 h. Then, the 20 boxes (each housing one spider) were placed beneath loose driftwood at Centennial Beach (Fig. 2a, c), and two spiders were randomly assigned to each of 10 pairs. All spiders were provisioned with *Zophobas morio* tenebrionid beetles (Salomon 2011) (Petsmart, Phoenix, AZ, USA) once each month between January to April, biweekly between May to October, and again once each month in November and December. The monthly food supply was based on a previous study (Salomon 2011). To quantify sex pheromones, the entire webs produced by the two spiders in each pair were collected at the end of each month and extracted overnight at 22 ºC in acetonitrile (400 µL). The samples were then stored at -20 ºC prior to pheromone analyses. Courtship-inducing contact pheromone components **1** and **7**, and their hydrolysis products **13** and **14**, were quantified based on a calibration curve established using synthetic **13** and **14** at 5 ng/µL, 2.5 ng/µL, 0.5 ng/µL, 0.25 ng/µL, and 0.05 ng/µL (Fischer et al. 2023a). Compound **14** was first detected in 2023, prompting us to re-analyse web extract samples of the year-long field experiment (2021) and to quantify **14**. The mate-attractant pheromone component **10** – which readily disseminates from webs – was quantified based on the amounts of **13** and **14** (as proxies for **10)** which accumulate on webs in identical stoichiometric ratios (Fig. 1) (Fischer et al. 2022). Daylength, temperature and precipitation data for Tsawwassen Beach were obtained from Environment and Natural Resources Canada (Government of Canada 2021). The field experiment was terminated on 31 December 2021 when mean and minimum overnight temperatures plummeted to − 22 ºC and − 45 ºC, respectively (see suppl. data for detailed weather data (Government of Canada 2021), killing all test spiders and many other wildlife on Centennial Beach (AF, personal observation).

### Statistical Analyses

Data were analysed using R (R Core Team 2024). Courtship occurrence by males was analysed using Fisher’s exact tests, whereas time spent courting by males in response to test stimuli was compared using a Whitney-Mann-U-test or a Kruskal-Wallis test. Capture data of male spiders in pheromone-baited traps (Exp. 7) were analysed using a one-sided binomial test (Fischer et al. 2022).

Seasonal variation in the amounts of contact pheromone components **1** and **7**, and their hydrolysis products **13** and **14**, on female webs over the course of a year (Exp. 8) was analysed using a Generalized Linear Mixed Effect Model (glmmTMB) following either a Gamma or a tweedie distribution (Brooks et al. 2017; Fischer et al. 2023a). Pairs of spiders nested in ‘month’ were set as a random factor to correct for repeated (monthly) pheromone quantifications, and fixed factors were (*i*) month of year, (*ii*) daylength, (*iii*) temperature, and (*iv*) precipitation. To determine the factor exerting the strongest effect on pheromone abundance, we used a model comparison approach (DiRienzo et al. 2019) for each log-transformed pheromone component, resulting in 16 models each with a single fixed factor. A more complex model structure was not considered because of the modest sample size of 10 observations for each month. Model assumptions were checked using the DHARMa package (Hartig 2022). A Type III ANOVA of the ‘car’ package (ANOVA) was used to test for significance of each model against the null model (Fox et al. 2019). Model comparisons were based on minimal Akaike’s Information Criteria (AIC) with small sample correction (Akaike 1987).

## Results

### H1a, b: Female *L. hesperus* Deposit Multiple Contact Pheromone Components on their Webs that Hydrolyse to Mate-Attractant Pheromone Components

#### HPLC-MS Analyses of Web Extracts

**Fig. 3:**
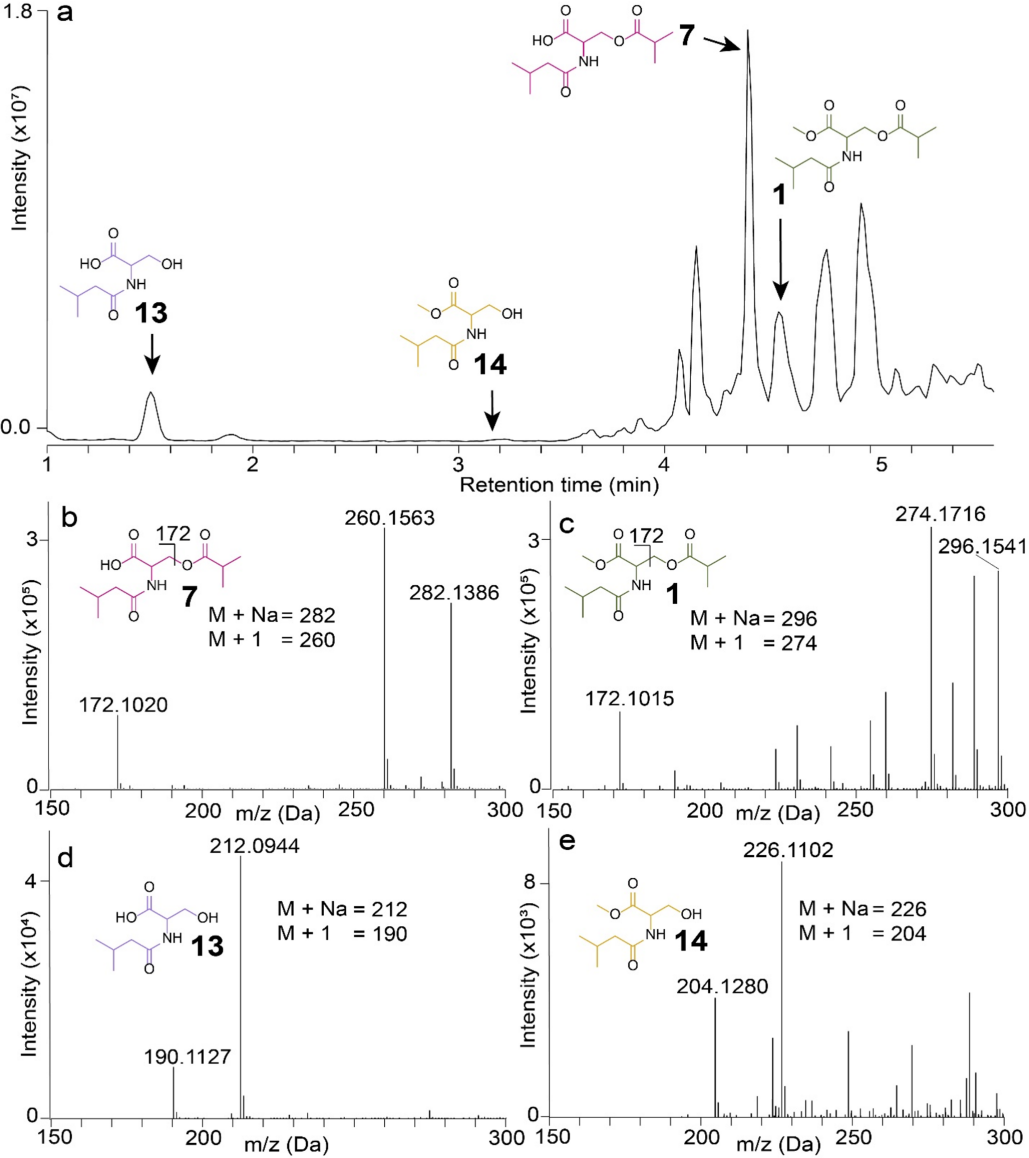
Total ion chromatogram (TIC) and positive electrospray ionization (+ ESI) spectra of (**a**) web extract of female *Latrodectus hesperus* analysed by high performance liquid chromatography-mass spectrometry, and (**b-e**) + ESI mass spectra of *N*-3-methylbutanoyl-*O*-isobutanoyl-L-serine (**7**) (**b**), *N*-3-methylbutanoyl-*O*-isobutanoyl-L-serine methyl ester (**1**) (**c**), *N*-3-methylbutanoyl-L-serine (**13**) (**d**), and *N*-3-methylbutanoyl-L-serine methyl ester (**14**) (**e**)

HPLC-MS analyses of web extracts revealed four candidate contact pheromone components (Fig. 3a, S1-14): (*i*) the previously reported component **1** (Scott et al. 2015b) with retention time (RT) 4.58 min and fragment ions 296.1541 (M + Na), 274.1716 (M + 1) and 172.1015 (Fig. 3c); (*ii*) the corresponding acid **7** with RT 4.37 min and fragment ions 282.1384 (M + Na), 260.1562 (M + 1) and 172.1018 (Fig. 3b); (*iii*) the hydrolysis product **13** (originating from **7**) with RT 1.50 min and fragment ions 212.0944 (M + Na) and 190.1124 (M + 1) (Fig. 3d); and (*iv*) the hydrolysis product **14** (originating from **1**) with RT 3.15 min and fragment ions 226.1102 (M + Na) and 204.1281 (M + 1) (Fig. 3e). GC-MS analyses of the corresponding trimethylsilyl derivatives of these components, and of authentic standards, confirmed the structural assignments (Figs. S15-19).

#### T-rod Bioassays of Contact Pheromone Components

**Fig. 4:**
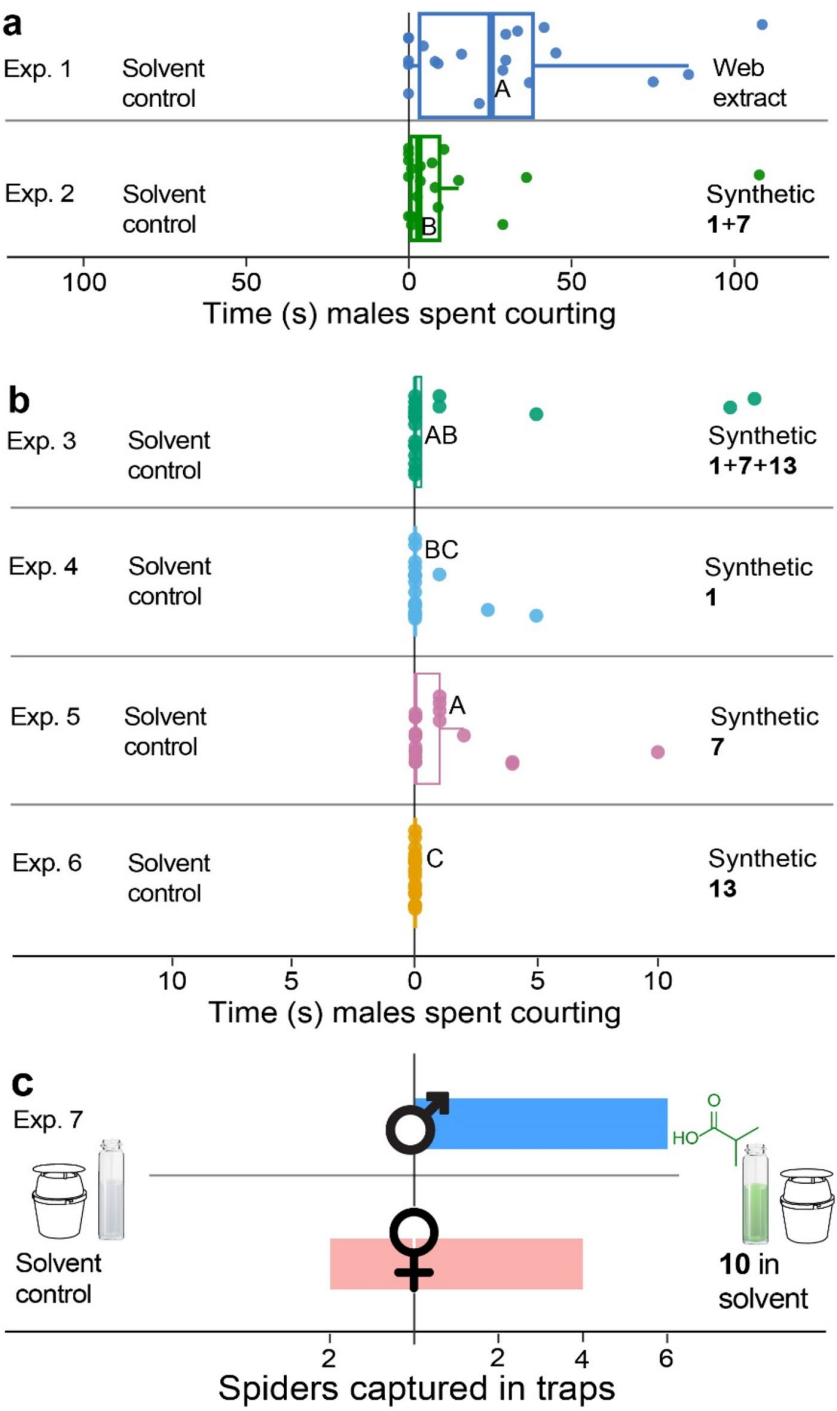
Behavioral responses of male *Latrodectus hesperus* to candidate contact (courtship-inducing) and mate-attractant pheromone components. (**a**, **b**) Duration (s) of courtship behaviour on a T-rod apparatus exhibited by *L. hesperus* males in response to (**a**) web extract of female *L. hesperus* or synthetic contact pheromone components **1** (*N*-3-methylbutanoyl-*O*-methylpropanoyl-L-serine methyl ester) and **7** (*N*-3-methylbutanoyl-*O*-methylpropanoyl-L-serine) at 1 web equivalent, and (**b**) synthetic **1**, **7**, and **13** (*N*-3-methylbutanoyl-L-serine) tested in a ternary blend at one web equivalent (**1** (47 ng), **7** (888 ng), **13** (65 ng); total = 1 µg) and singly at 1 µg each. (**c**) Field captures of male and female *L. hesperus* in Unitraps (Fig. 1) baited with **10** (the synthetic mate-attractant pheromone component isobutyric acid). Different letters in subpanel **a** (Whitney-Mann-U-test), and in subpanel **b** (Kruskal-Wallis test), denote statistical differences (*p* < 0.05); in subpanel **c**, more males were captured in traps baited **10** than in control traps (*p* = 0.016, binomial test)

Web extract of adult unmated female *L. hesperus*, applied to filter paper, did elicit courtship by 15 out of 20 males, whereas solvent controls did not (Exp. 1: *p* < 0.001, Fisher’s exact test). Identical results were obtained when the synthetic blend of contact pheromone components **1** and **7** was tested *versus* a solvent control (Exp. 2: *p* < 0.001, Fisher’s exact test). Consequently, there was no statistical difference in the occurrence of courtship by males in response to web extract and to synthetic **1** and **7** (*p* = 1, Fisher’s exact test). However, males courted longer on filter paper treated with web extract than on filter paper treated with synthetic **1** and **7** (W = 273, *N* = 20, *p* = 0.048, Exp. 1 + 2, Fig. 4a), indicating the potential presence of additional unidentified contact pheromone components in web extract.

Synthetic components **1**, **7** and **13**, tested singly and in ternary combination, differed in their ability to induce and maintain courtship by males (Exps. 3–6: χ² = 9.96, df = 3, *p* = 0.020, Kruskal-Wallis test, Fig. 4b), with solvent controls invariably not eliciting any courtship. In response to **7**, and to the ternary blend of **1**, **7** and **13**, males courted equally long (z = -0.99, *p* = 0.160), but **7** prompted longer courtship than **1** (z = 1.86, *p* = 0.031) and **13** (z = 3.03, *p* = 0.001). There were no statistical differences between the males’ responses to (*i*) **1** and the ternary blend of **1**, **7** and **13** (z = 0.87, *p* = 0.193), and (*ii*) **1** and **13** (z = − 1.17, *p* = 0.121). Component **13** did not elicit courtship by males. Component **14** had not been found at the time of bioassays.

#### Field Testing of the Candidate Mate-Attractant Pheromone Component Isobutyric Acid

Traps baited with synthetic isobutyric acid (**10**) – the candidate mate-attractant pheromone component originating from **1** to **7** (Fig. 1) – captured six males, whereas unbaited control traps captured none (*p* = 0.016, binomial test, Fig. 4c). Interestingly, traps baited with **10** also captured four mature females, whereas unbaited control traps captured two (*p* = 0.688, binomial test, Fig. 4c).

**Fig. 5:**
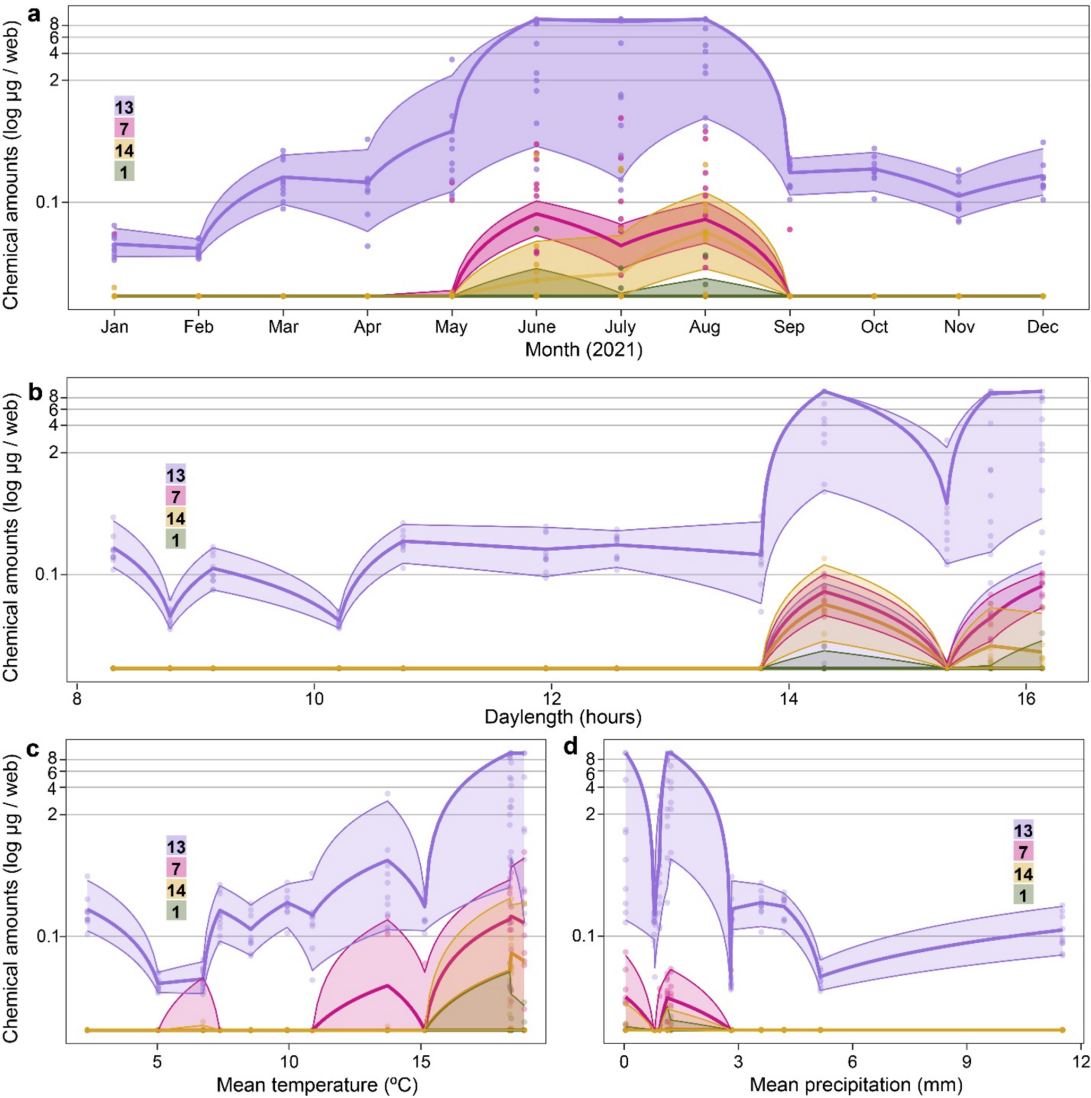
(**a**) Amounts of contact pheromone components **7** (*N*-3-methylbutanoyl-*O*-methylpropanoyl-L-serine) and **1** (*N*-3-methylbutanoyl-*O*-methylpropanoyl-L-serine methyl ester), and their hydrolysis products **13** (*N*-3-methylbutanoyl-L-serine) and **14** (*N*-3-methylbutanoyl-L-serine methyl ester), extracted every month (2021) from webs of female *L. hesperus* kept in wooden boxes (Fig. 1d) at Centennial Beach (the spider collection site). (**b-d**) Amounts of **7**, **1**, **13** and **14** in relation to mean daylength (photophase) (**b**), temperature (**c**), and mean precipitation (**d**). Dots represent recorded data, whereas lines and shaded regions are back-transformed model predictions with 95% confidence intervals from generalized linear mixed effect models (GLMMs)

### H2: Female *L. hesperus* Increase Pheromone Signalling During the Primary Mating Season

The amount of contact pheromone components **1** and **7** that unmated adult female *L. hesperus* deposited on their webs varied over the course of a year (Fig. 5; Table 1). Both **1** and **7** were detected on webs only during the summer months (Fig. 5a). As the corresponding hydrolysis products **13** and **14** accumulated on webs year-round and peaked during the summer months, it follows that the mate-attractant pheromone component **10** (Fig. 4c) disseminated from webs in a corresponding pattern.

All factors (month of year, daylength, temperature, precipitation) produced significant models for pheromone components **1** and **7**, and hydrolysis products **13** and **14**. Consistently, ‘month of year’ and ‘daylength’ were the best fit factors with highest values during the long photophase of the summer months (see AIC values in Table 1; Fig. 5a + b). ‘Temperature’ was the second-best fit factor, revealing elevated amounts of **1** and **7**, and of **13** and **14**, with higher temperature (Fig. 5c; Table 1). Precipitation was the third-best fit factor (Table 1), revealing an inverse relationship between the amount of precipitation and the quantity of pheromone (Fig. 5d).

**Table 1:**
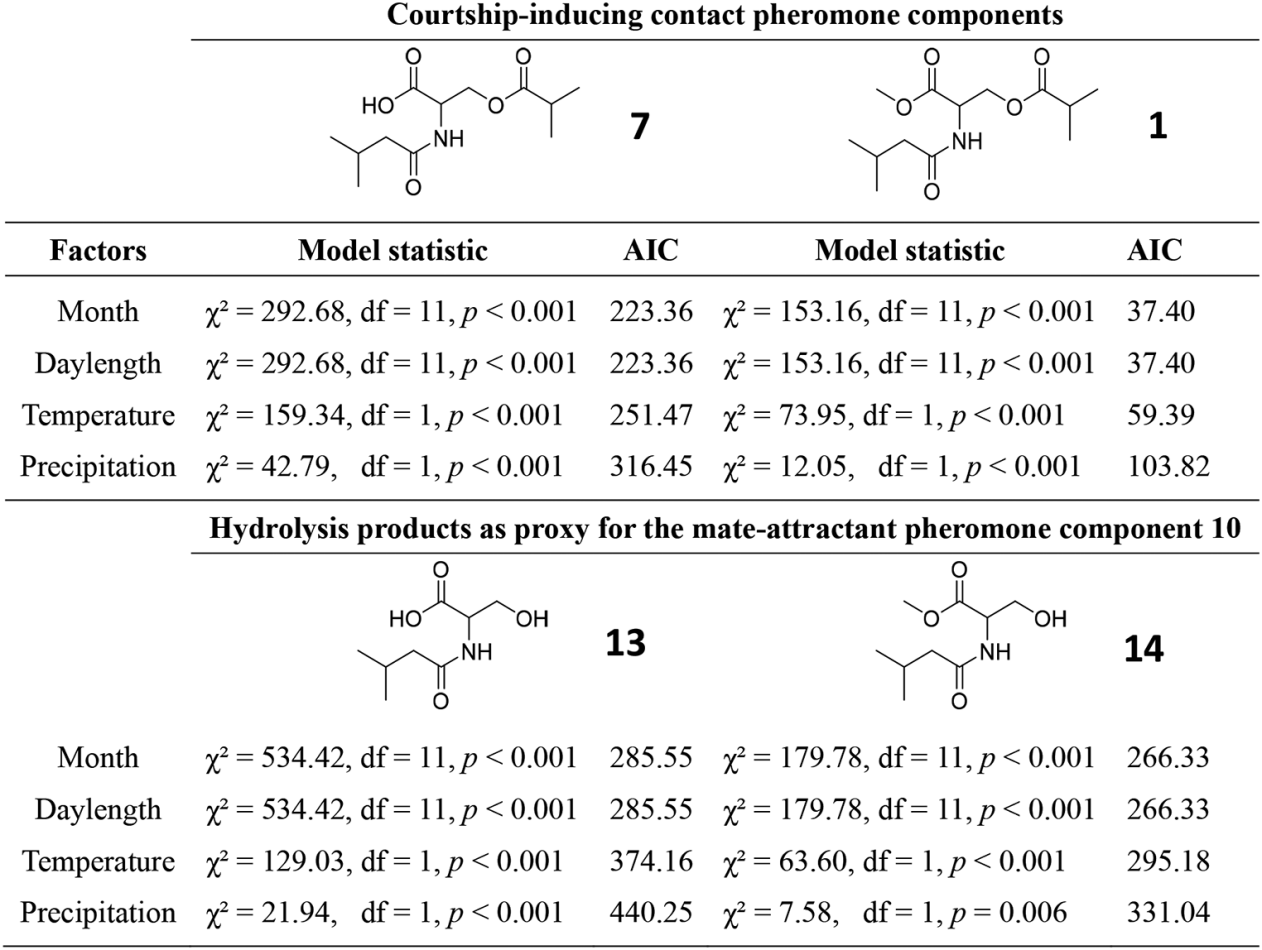
Statistical results of generalized mixed effect models (GLMMs, Exp. 8). Quantities of (*i*) the pheromone components *N*-3-methylbutanoyl-*O*-methylpropanoyl-L-serine (**7**) and *N*-3-methylbutanoyl-*O*-methylpropanoyl-L-serine methyl ester (**1**), and (*ii*) the hydrolysis products of **7** [*N*-3-methylbutanoyl-L-serine (**13**)] and of **1** [*N*-3-methylbutanoyl-L-serine methyl ester (**14**)] were analysed using various factors and best fit model selections based on minimal Akaike’s information criteria (AIC) for each model group

## Discussion

Our study revealed *N*-3-methylbutanoyl-*O*-methylpropanoyl-L-serine (**7**) as a previously unknown but behaviourally important courtship-inducing contact pheromone component of female *L. hesperus.* Both **7** and the previously known contact pheromone component *N*-3-methylbutanoyl-*O*-methylpropanoyl-L-serine methyl ester (**1**) (Scott et al. 2015b) hydrolyse at the ester bond, releasing the mate-attractant pheromone component isobutyric acid (**10**) (Fig. 1). Female *L. hesperus* deposited significantly larger amounts of **7** and **1** during the warm summer months than in the cooler and wetter spring and fall, revealing seasonality of sexual signalling which aligns with the greater availability of sexually mature males during the summer.

### H1a: Female *L. hesperus* Deposit Multiple Contact Pheromone Components on their Webs

The synthetic blend of **7** and **1** elicited courtship in as many males as did web extract, indicating that this blend is more bioactive than just **1** which prompted courtship in only a few males (Scott et al. 2015b; this study). The identification of **7** required an analytical approach that entailed both HPLC-MS analysis of crude web extract and GC-MS analysis of BSTFA-derivatized web extract. Compound **13** – the hydrolysis product of **7** (Fig. 1) – did not elicit male courtship, nor did hydrolysis product **12** of the contact pheromone components **4**, **5** and **6** produced by female *S. grossa* (Fig. 1) (Fischer et al. 2022). Drawing on both the *S. grossa* data and on findings in this study that the hydrolysis product **13** does not elicit courtship by males (Fig. 4b), we infer (but have yet to experimentally test) that **14**, the hydrolysis product of **1** (Fig. 1), does likewise not induce any courtship by males. Compound **14** was found only after laboratory bioassays of **1**, **7**, and **13** were completed. However, we did find and quantify **14** in the context of the year-long field experiment of 2021 (Fig. 5). Putative unknown component(s) in web extract that prolonged the males’ courtship relative to the ternary blend of **1**, **7**, and **13** (Fig. 4a) may be discovered using non-targeted metabolomics, which have proven effective in searches for new (spider) pheromone components (Liu et al. 2022; Fischer et al. 2023b). Interestingly, **7** was only recently discovered as a minor pheromone component in the triangulate cobweb spider, *Steatoda triangulosa* (Fischer et al. 2023b).

### H1b: Contact Pheromone Components Hydrolyse to Mate-Attractant Pheromone Components

As both *N*-3-methylbutanoyl-L-serine (**13**) and *N*-3-methylbutanoyl-L-serine methyl ester (**14**) – the hydrolysis products of **7** and **1**, respectively (Fig. 1b) – were present on webs, we hypothesized that isobutyric acid (**10**) – also originating from **7** and **1** (Fig. 1) – is the mate-attractant pheromone component that emanates from webs of female *L. hesperus*. The hypothesis was supported in a field experiment, demonstrating that traps baited with synthetic **10** captured *L. hesperus* males. Although captures of six *L. hesperus* males in a single night exceeded our expectations, it is still conceivable that there are additional, as yet unknown, mate-attractant pheromone component that would have enhanced the attractiveness of the trap lure.

Transition of courtship-inducing contact pheromone components to airborne mate-attractant pheromone components, as demonstrated in our study, was first discovered in the linyphiid spider *Linyphia triangularis* (Schulz and Toft 1993). As the mate-attractant pheromone component **10** was released at levels too low for detection and quantification, we inferred quantitative emission of **10** from webs using the amounts of web-borne hydrolysis products **13** and **14** as proxys, essentially taking the same approach as in studies with *S. grossa* (Fischer et al. 2022, 2023a, 2024) and *S*. *triangulosa* (Fischer et al. 2023b). It is noteworthy that **13** and **14** were hardly detectable in web extracts of 3-day-old webs, whereas large quantities of **13** and **14** were detected in extracts of 30-day-old webs collected in the field experiment (H2, see Fig. 5). It seems that within 30 days most of the deposited contact pheromone components **1** and **7** transition to the mate-attractant pheromone component **10**. It follows that female spiders continuously deposit new contact pheromone components onto their web, thereby remaining attractive to males.

Sustained dissemination of mate-attractant pheromone components from a reservoir of web-borne contact pheromone components is a unique mechanism of sexual signalling (Fischer et al. 2022) in sessile web-building spiders (Janetos 1982). Sustained pheromone dissemination from the permanent web of cobweb spiders continuously informs potential signal recipients about the ‘fixed’ location of a receptive female. Unlike many vagabonding insects which must actively emit pheromone to update prospective mates about their ever-changing location (Levi-Zada and Byers 2021), female cobweb spiders remain on their webs, depositing contact pheromone components which transition to mate-attractant pheromone components. This transition is thought to be mediated by a hydrolase enzyme present on webs (Fischer et al. 2022). The hydrolysis rate is positively correlated with the pH of the spider’s silk, ranging from pH 4 to 7 (Fischer et al. 2022). If we were to accept the premise that spider females are able to modify their web’s pH, it follows that they can adjust the dissemination rate of mate-attractant pheromone. Spider females may modify their silk’s pH during the ‘acid bath’ of silk fiber production (Vollrath et al. 1998; Heim et al. 2009) or via phosphate pH buffers (Schildknecht et al. 1972). Although the mechanisms underlying release rate adjustments of mate-attractant pheromone are not fully understood, evidence is mounting that females are capable of these adjustments. For example, starving (and thus infecund) *S. grossa* females deposited less contact pheromone onto their webs but remained as attractive to males as fed (and thus fecund) females by increasing the release rate of mate-attractant pheromone (Fischer et al. 2024). Similarly, in response to perceived mate-competition, female *S. grossa* increased the release rate of mate-attractant pheromone (Fischer et al. 2023a). Likewise, female *L. hesperus* may be able to adjust release rates of their mate-attractant pheromone components. Upon arrival on a web, males engage in courtship, cutting and bundling up sections of the female’s web while adding their own silk (Scott et al. 2012, 2015a). The resulting diminished attractiveness of the female’s web to rival males is due to altered release dynamics of the modified web and/or pheromone emanation from the male’s deposited silk.

There is tantalizing (but statistically not significant) evidence (due to small sample size) that female *L. hesperus* may recognize and respond to their own sex pheromone (pheromone autodetection (Holdcraft et al. 2016; Suresh et al. 2023). Traps baited with synthetic isobutyric acid (**10**) captured four female *L. hesperus* whereas unbaited control traps captured two. That female *L. hesperus* near conspecific females are less likely to relocate their webs (Salomon 2009) may be attributed, in part, to their sensing of isobutyric acid (**10**), the mate-attractant sex pheromone component. If true, this would help explain why webs of female *L. hesperus* occur in clusters (Salomon et al. 2010). Clustered female *L. hesperus* attract prospective mates faster, or more effectively, than solitary females (Scott et al. 2019, 2020), possibly suggesting cooperative mate-calling activities in this promiscuous species. Yet, when *S. grossa* females were surrounded by other females, they deposited larger amounts of contact pheromone components on their webs, and they accelerated the transition of contact pheromone components to mate-attractant pheromone components, indicative of perceived competition for mates (Fischer et al. 2023a). Conversely, females of the orb weaver *Araneus diadematus* avoid the odor of conspecific females (Fischer et al. 2021). In *L. hesperus*, pheromone autodetection may also inform social behavior. During the wet and cold winter months, females share webs but resume their solitary lifestyle when the mating season approaches (Salomon et al. 2010) and their pheromone signalling intensifies (Fig. 5). It is also conceivable that females may respond to isobutyric acid not in recognition of a communication or social signal but in recognition of a prey cue because isobutyric acid is common in secretions of potential prey (El-Sayed 2025).

### (H2) Females Increase Pheromone Signalling During the Primary Mating Season in the Summer

In line with the ‘strategic-signalling hypothesis’ that animals aim to optimize the cost/benefit ratio of sexual signalling (Weiss and Schneider 2022), female *L. hesperus* adjusted pheromone signalling in accordance with the availability of mates. Females elevated pheromonal signalling during the mating season in the summer when most males are searching for mates (Salomon et al. 2010). Indeed, the courtship-inducing contact pheromone components **1** and **7** – which release the mate-attractant pheromone component isobutyric acid (**10**) during hydrolysis – were detectable only during the summer. Nonetheless, it seems that females remain at least moderately attractive to males even outside the main mating season (Fig. 5). This interpretation is based on web extract analysis data showing that **13**, the hydrolysis product of **7**, was detectable year-round, implying that **7** has been deposited on webs, and **10** emanates from webs, year-round. The same concept can be applied to **14** and **1**. That neither **7**, nor **14** and **1**, were detectable in web extracts obtained outside the main mating season was likely due to their occurrence at levels below the detection threshold of our instruments. That females continued to deposit contact pheromone components in spring and fall – albeit in much lower amounts – indicates a trade-off between energy savings for pheromone biosynthesis and additional mating opportunities with ‘off-season’ males. As daylength (length of photophase) and month of year were the best model predictors for the amount of contact pheromone components and their hydrolysis products on webs (Fig. 5, Table 1), it is conceivable that the increasing photophase in spring informs *L. hesperu*s females about an approaching mating season, thereby prompting them to deposit more pheromone on their webs. Conversely, the decreasing photophase in the fall may inform females that the main mating is nearing its end, thus prompting them to reduce signalling. The increase in pheromone deposition in the summer not only coincides with the presence of mature males, but also with rising temperatures (Government of Canada 2021) and greater prey availability (Salomon 2011) which may help offset costs for pheromone biosynthesis. As the pheromone components **7**, **1**, and **10** are water soluble, they are subject to wash-off by precipitation which would reduce the abundance of courtship-inducing **7** and **1**, and ultimately the release of the airborne mate-attractant pheromone component **10**. This would explain the inverse relationship between heavy rain and low abundance of **7** and **1** (which give rise to **10**) during the wet winter months.

Strategic signalling has been reported in laboratory experiments for unmated females of the wasp spider, *Argiope bruennichi.* As they age and approach obligatory oviposition, female wasp spiders increase sexual signalling and produce more pheromone; conversely, females cease signalling following copulation (Weiss and Schneider 2022, 2023). Increasing attractiveness with age seems to be common among spiders (Fischer 2019). Our study reveals that annual seasonality is yet another, and apparently even stronger, determinant of sexual signalling than age in long-lived female *L*. *hesperus.* Extensively starved and infecund *S. grossa* females also engaged in strategic signalling in that they accelerated the conversion of contact pheromone components to mate-attractant pheromone components, effectively deceiving males that would gain reproductive fitness by selecting fecund females as mates (Fischer et al. 2024). Whereas *S. grossa* females endure extensive starvation without adverse physiological effects (Fischer et al. 2024), *L*. *hesperus* females are sensitive to starvation (Blackledge and Zevenbergen 2007) and starved females are less ‘appealing’ to males (Baruffaldi and Andrade 2015). Diminished pheromone production as a result of starvation or of signalling outside the main mating season [this study] implies significant pheromone production costs, an aspect that requires further investigation (Fischer et al. 2024).

## Conclusion

We report *N*-3-methylbutanoyl-*O*-methylpropanoyl-L-serine (**7**) as a previously unrecognized contact pheromone component of female *L. hesperus.* In binary combination, **7** and *N*-3-methylbutanoyl-*O*-methylpropanoyl-L-serine methyl ester (**1**), the previously reported contact pheromone component of female *L. hesperus* (Scott et al. 2015b), induced courtship in as many males as did web extracts, but web extract elicited more sustained courtship by males, indicating the presence of additional, still unknown, contact pheromone components. Both **7** and **1** hydrolyse at the isobutyrate ester bond, thus releasing the previously unknown mate-attractant pheromone component isobutyric acid (**10**). Female *L. hesperus* increased pheromone deposition and dissemination during the warm and dry summer months when sexually mature males are most abundant, providing evidence for strategic signalling.

## Electronic Supplementary Material

Below is the link to the electronic supplementary material.

Supplementary Material 1

Supplementary Material 2

Supplementary Material 3

## Data Availability

Data is provided within the manuscript or supplementary material.
