# Supplementary material for: Identification and Seasonal Abundance of Web- and Air-Borne Sex Pheromone Components of Western Black Widow Spiders, Latrodectus hesperus
Source: J Chem Ecol. 2025 Mar 12;51(2):36. doi: 10.1007/s10886-025-01590-6 (PMC11903603; doi:10.1007/s10886-025-01590-6)
Supplement: Supplementary file 3 — Supplementary Material 3 [file 10886_2025_1590_MOESM3_ESM.docx]

# **Supplementary Material**

# Identification and seasonal abundance of web- and air-borne sex pheromone components of western black widow spiders, *Latrodectus hesperus*

Andreas Fischer^1,2^*, Alexandra J. Fischer^1^, Regine Gries^1^, Emmanuel Hung^1^, Kelvin Lau^1^, Aryan Monfared^1^, Gerhard Gries^1^

^1^Department of Biological Sciences, Simon Fraser University, Burnaby, BC, Canada
^2^Department of General and Systematic Zoology, University of Greifswald, Greifswald,

Germany

*Corresponding author: andreas.fischer@uni-greifswald.de


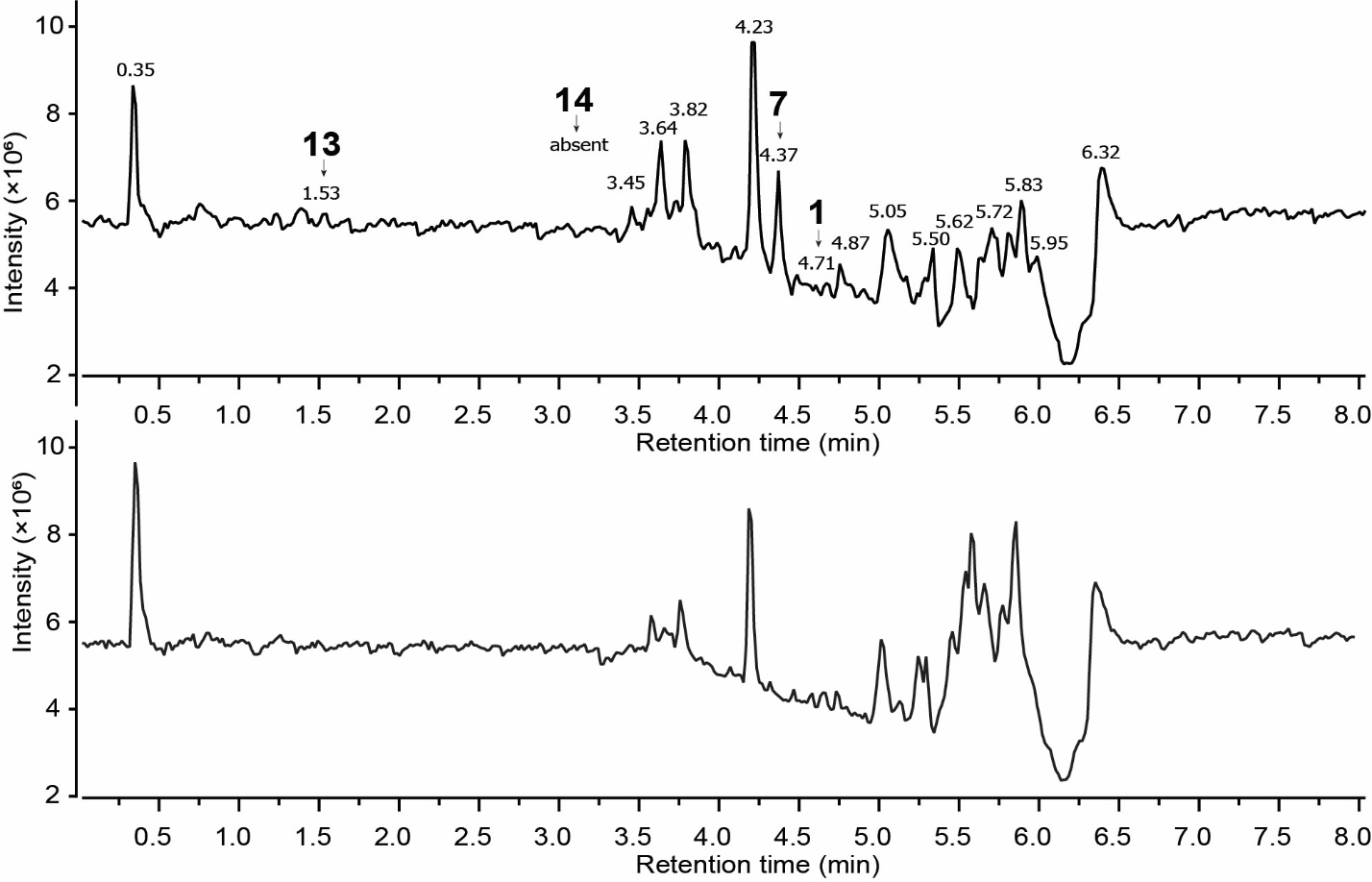
**Figure S1:** Total ion chromatograms of web extracts of adult unmated female (upper trace) and subadult unmated female (lower trace) *Latrodectus hesperus* analysed by high performance liquid chromatography-mass spectrometry. **1** = *N*-3-methylbutanoyl-*O*-isobutanoyl-L-serine methyl ester, **7** = *N*-3-methylbutanoyl-*O*-isobutanoyl-L-serine, **13** = *N*-3-methylbutanoyl-L-serine, **14** = *N*-3-methylbutanoyl-L-serine methyl ester. The mass spectra of **1**, **7**, **13** and **14** are reported in Figure 3 of the main text.


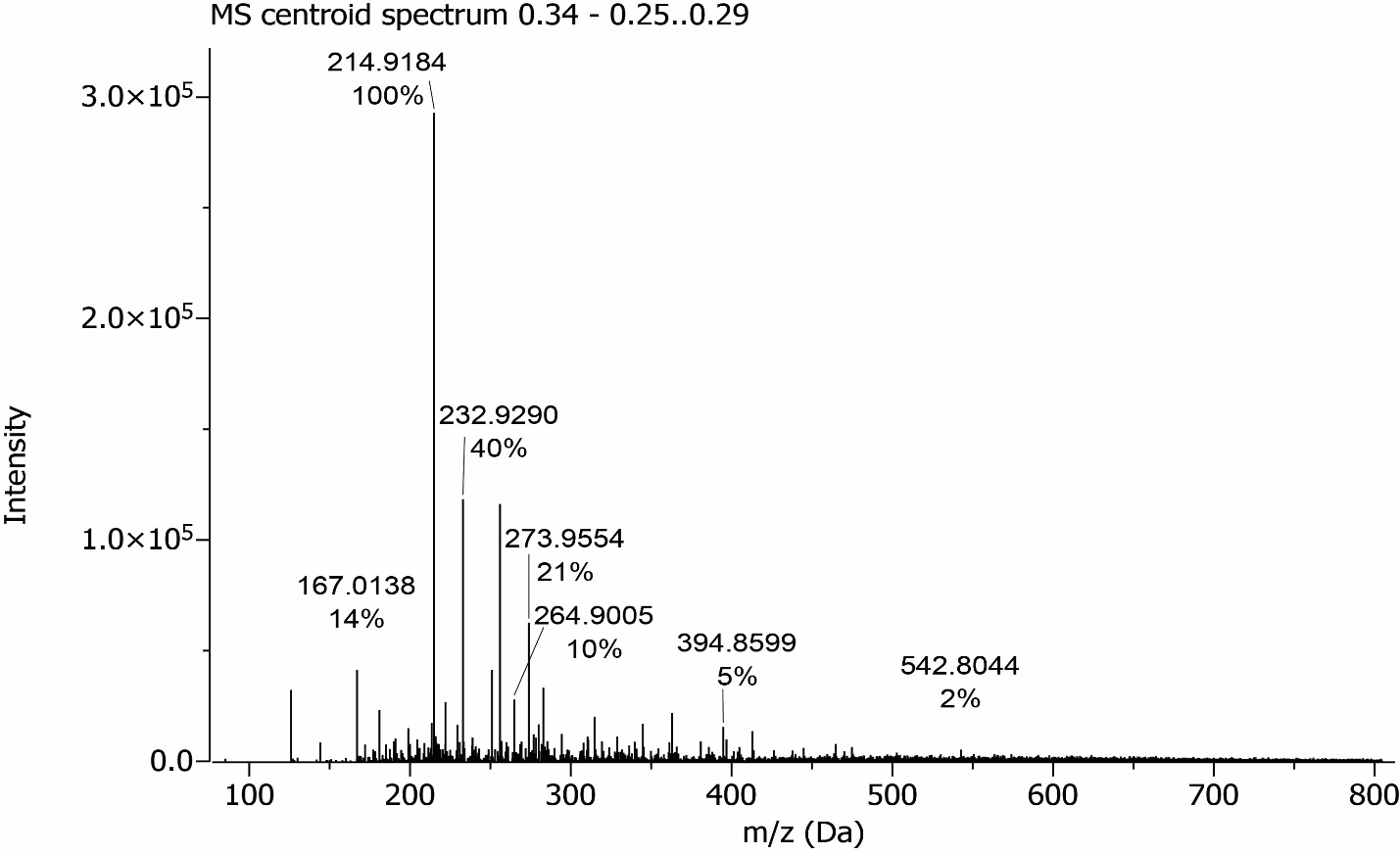


**Figure S2:** Positive electrospray ionization spectrum of a compound at retention time 0.35 min in web extract of adult female *Latrodectus hesperus* (Fig. S1) analyzed by high performance liquid chromatography-mass spectrometry.


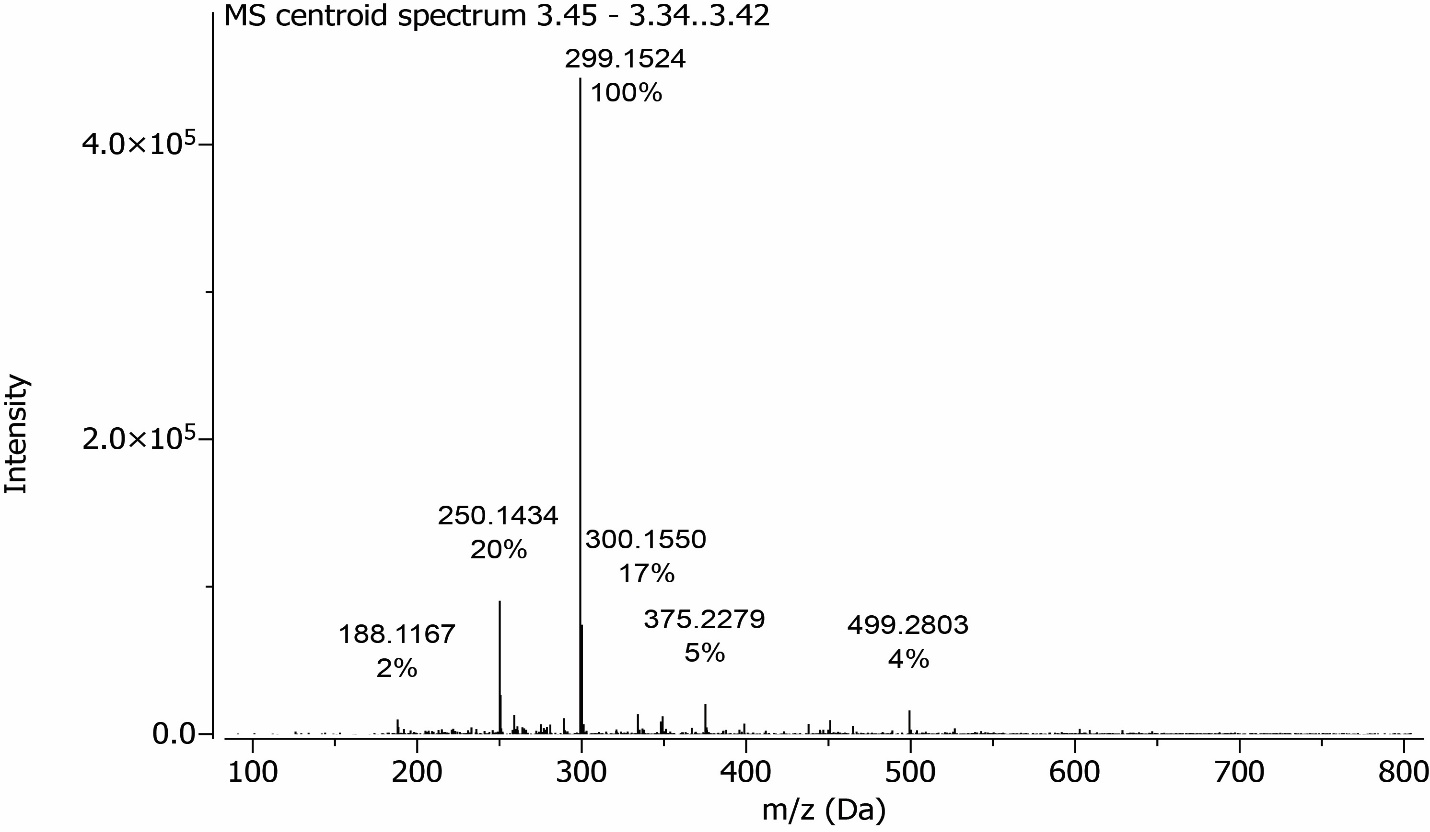


**Figure S3:** Positive electrospray ionization spectrum of the compound at retention time 3.45 min in web extract of adult female *Latrodectus hesperus* (Fig. S1) analyzed by high performance liquid chromatography-mass spectrometry.


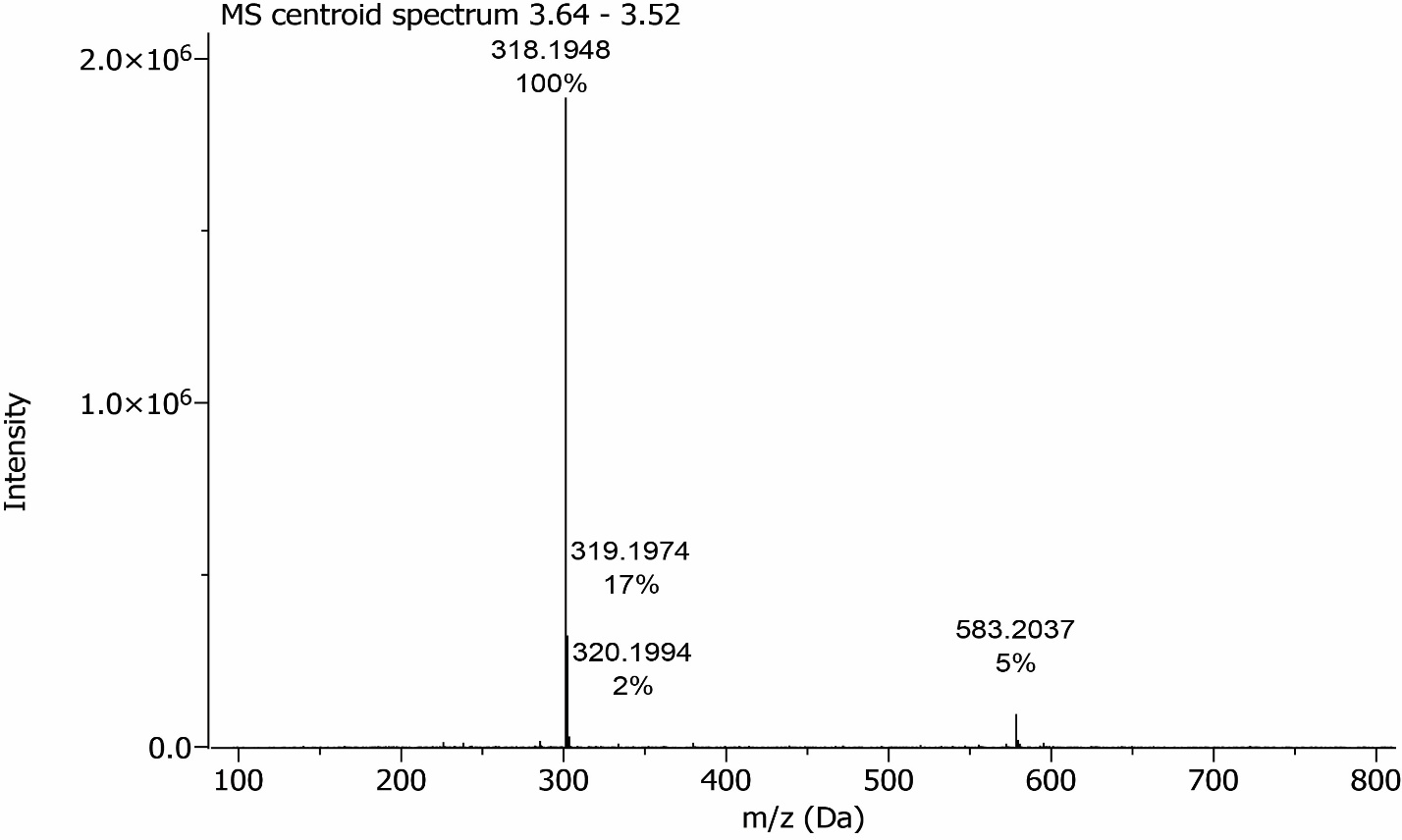
 **Figure S4:** Positive electrospray ionization spectrum of the compound at retention time 3.64 min in web extract of adult female *Latrodectus hesperus* (Fig. S1) analyzed by high performance liquid chromatography-mass spectrometry.


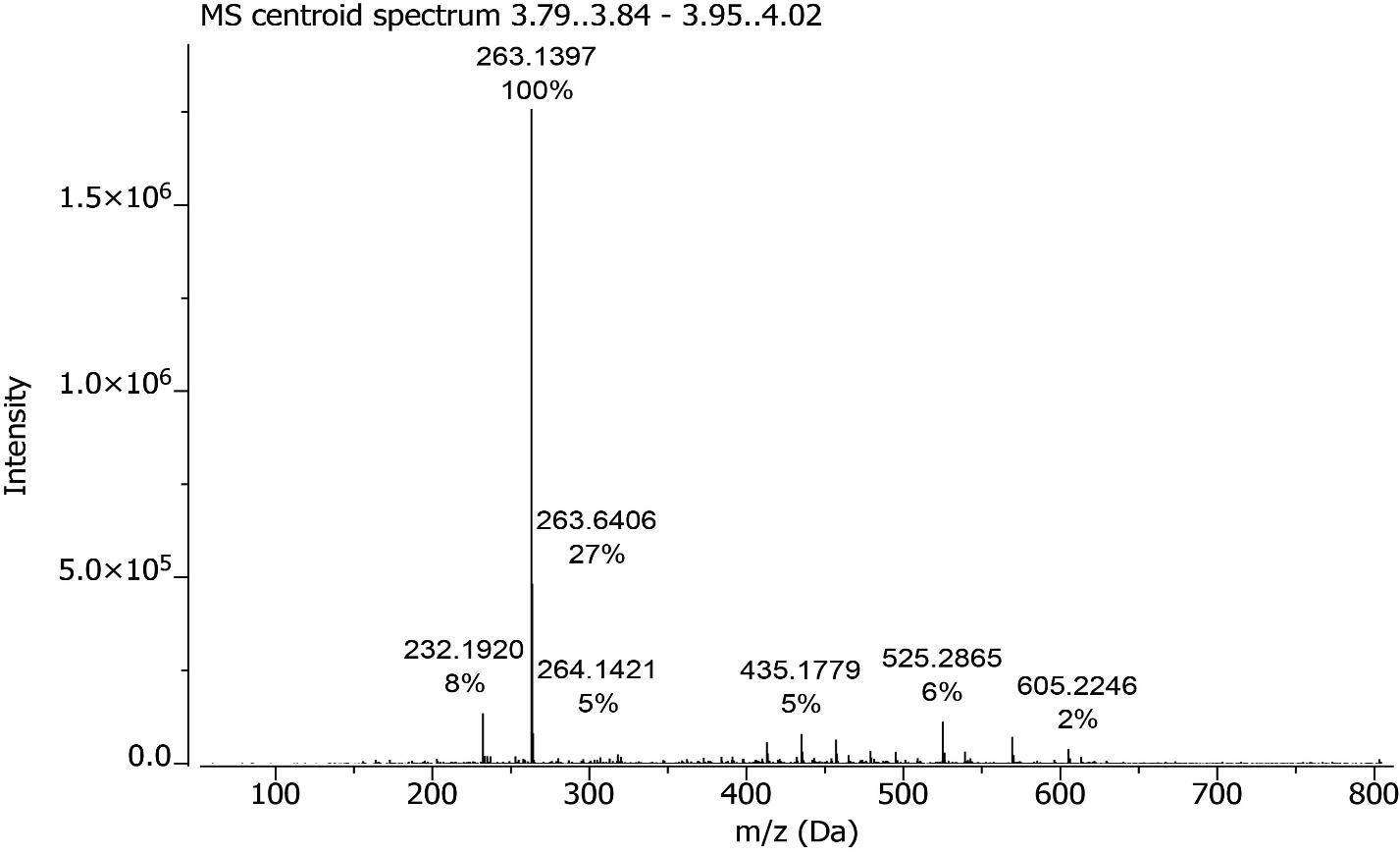


**Figure S5:** Positive electrospray ionization spectrum of the compound at retention time 3.82 min in web extract of adult female *Latrodectus hesperus* (Fig. S1) analyzed by high performance liquid chromatography-mass spectrometry.


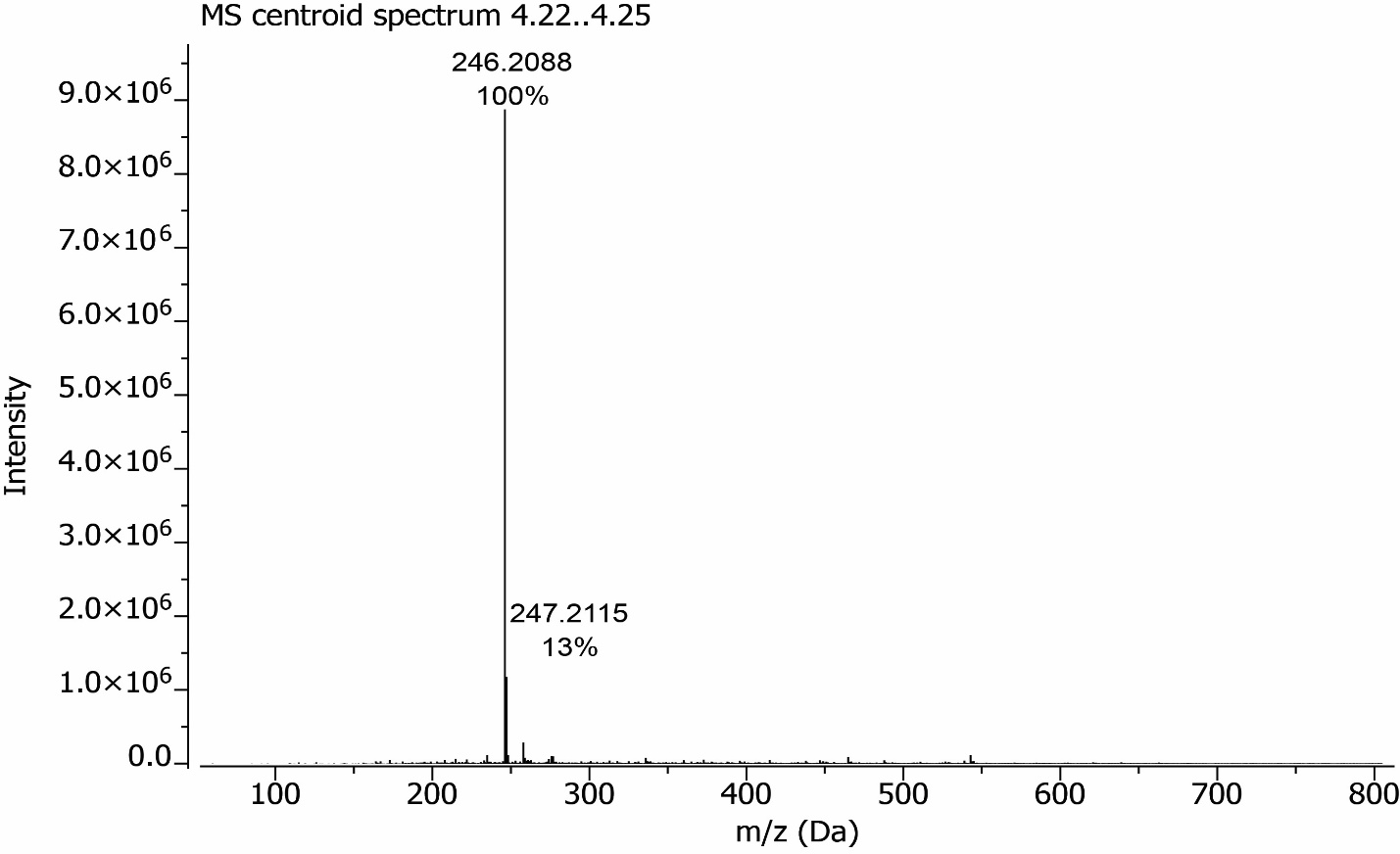


**Figure S6:** Positive electrospray ionization spectrum of the compound at retention time 4.23 min in web extract of adult female *Latrodectus hesperus* (Fig. S1) analyzed by high performance liquid chromatography-mass spectrometry.


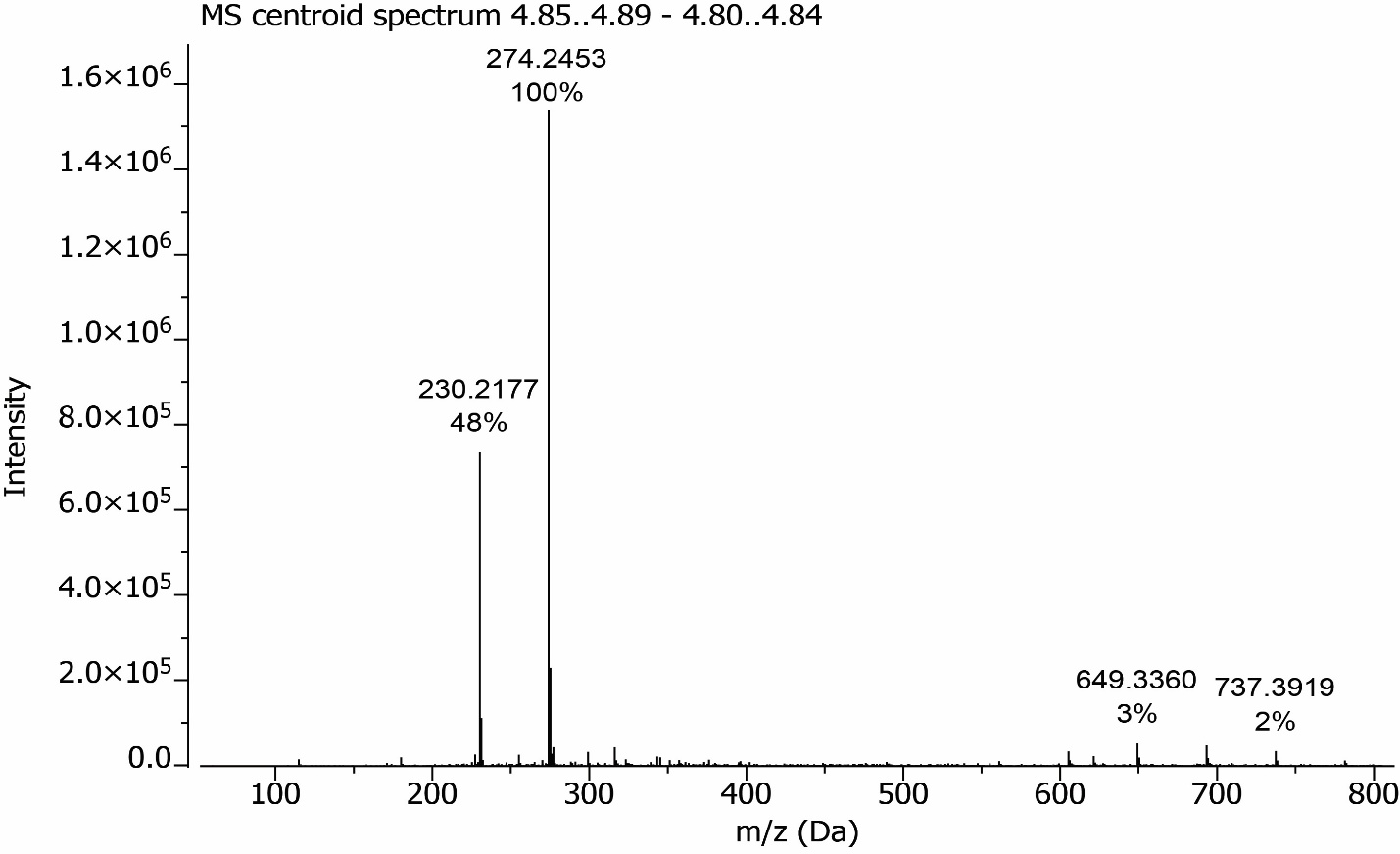


**Figure S7:** Positive electrospray ionization spectrum of the compound at retention time 4.87 min in web extract of adult female *Latrodectus hesperus* (Fig. S1) analyzed by high performance liquid chromatography-mass spectrometry.


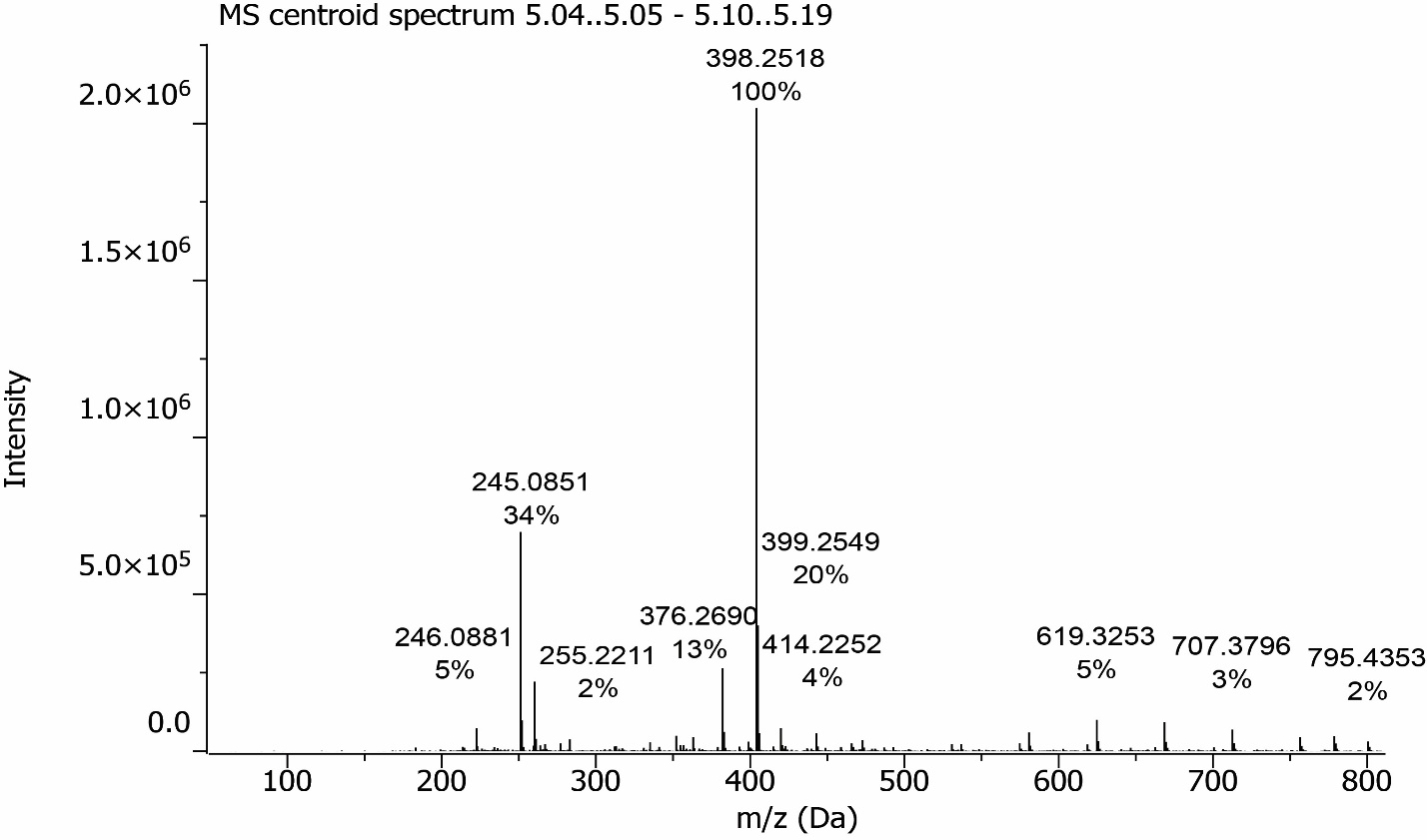


**Figure S8:** Positive electrospray ionization spectrum of the compound at retention time 5.05 min in web extract of adult female *Latrodectus hesperus* (Fig. S1) analyzed by high performance liquid chromatography-mass spectrometry.


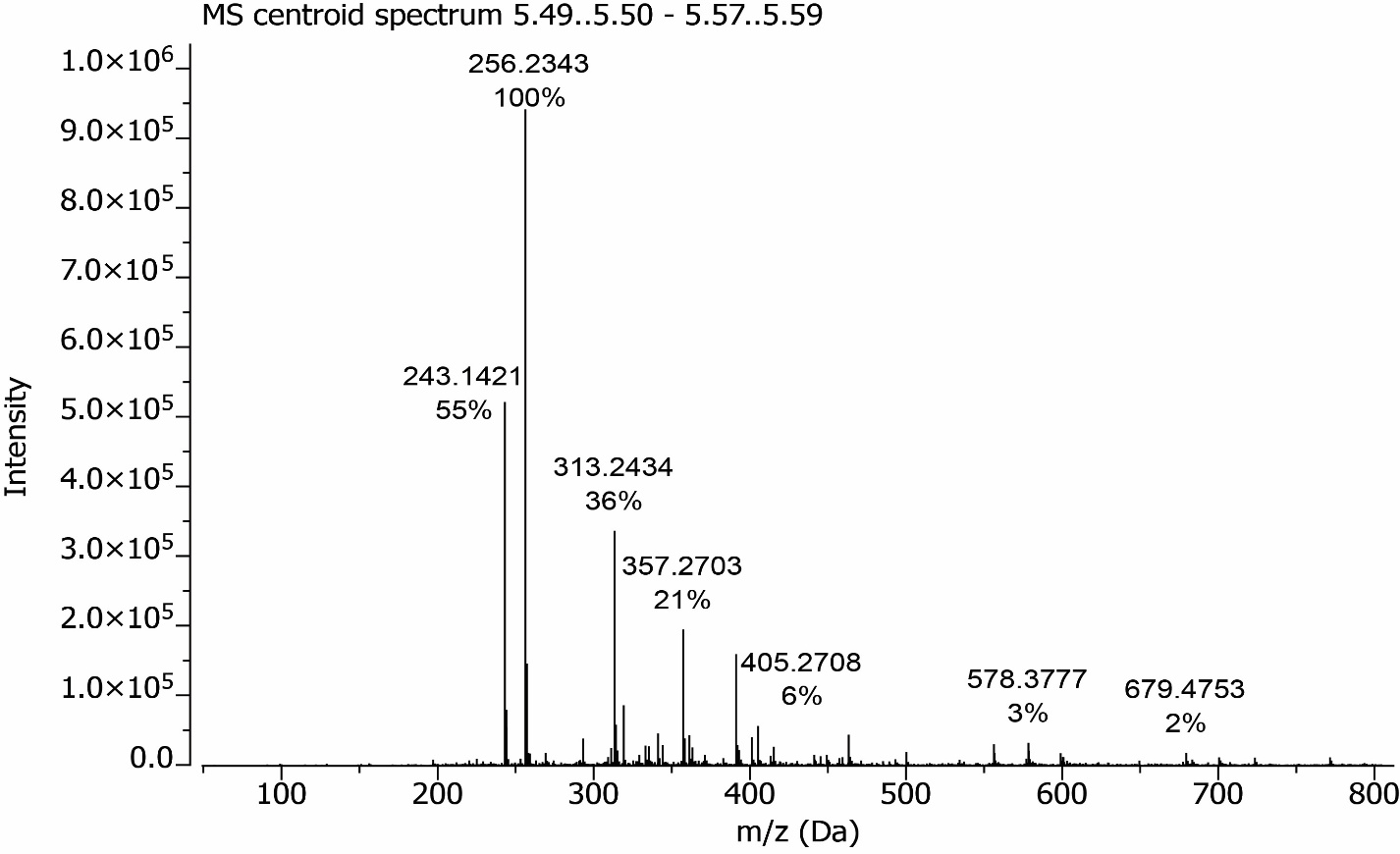


**Figure S9:** Positive electrospray ionization spectrum of the compound at retention time 5.50 min in web extract of adult female *Latrodectus hesperus* (Fig. S1) analyzed by high performance liquid chromatography-mass spectrometry.


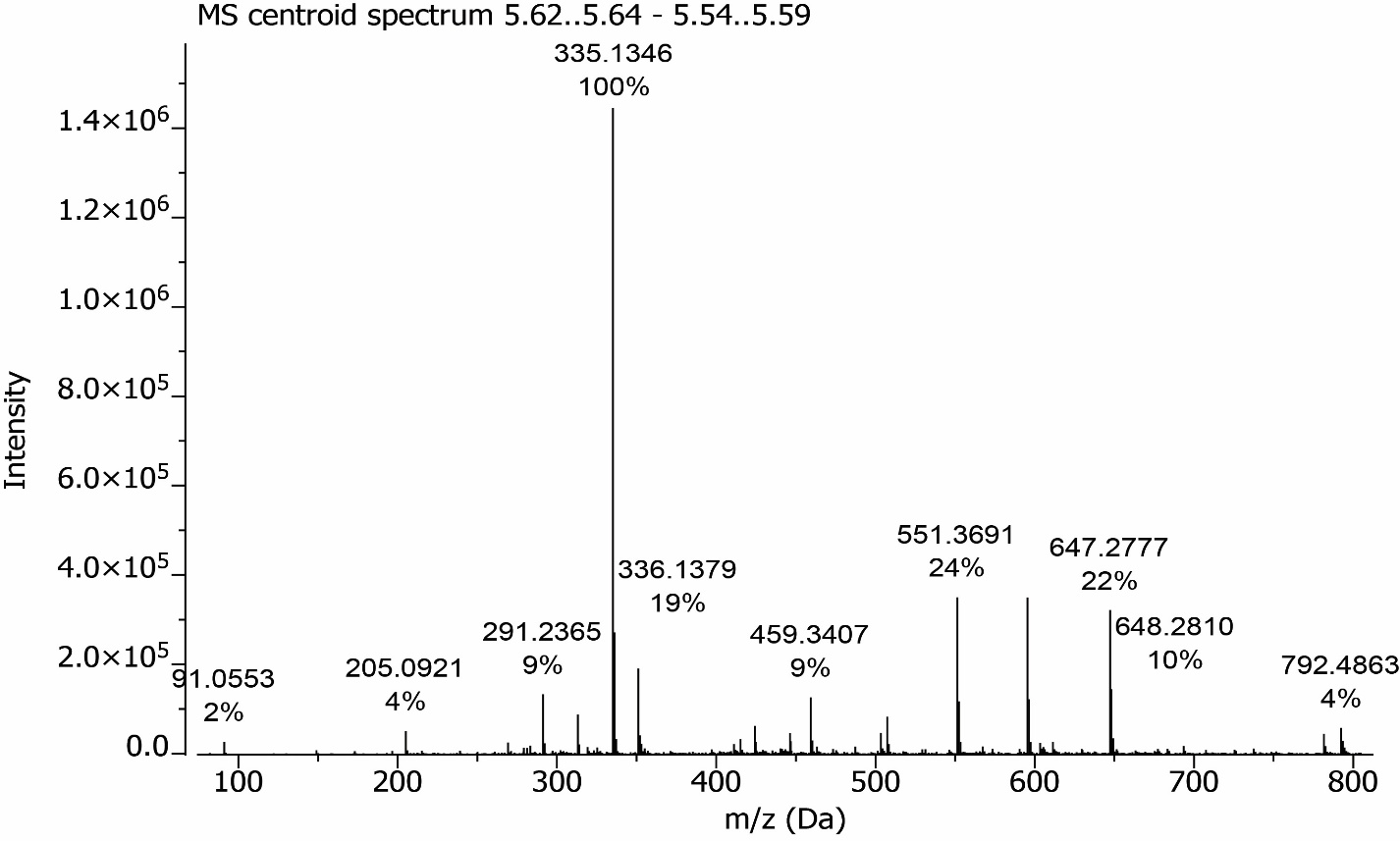


**Figure S10:** Positive electrospray ionization spectrum of the compound at retention time 5.62 min in web extract of adult female *Latrodectus hesperus* (Fig. S1) analyzed by high performance liquid chromatography-mass spectrometry.


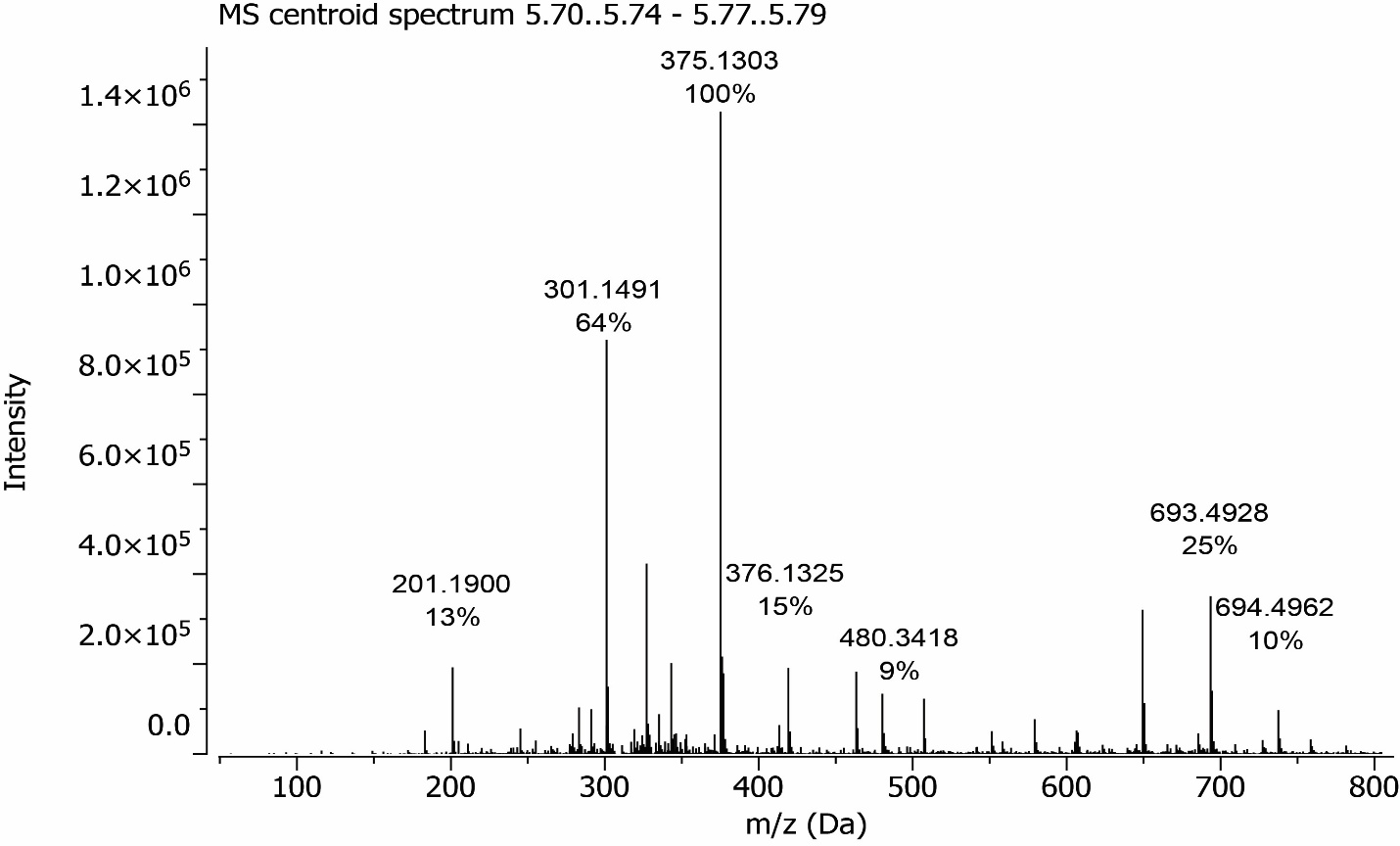


**Figure S11:** Positive electrospray ionization spectrum of the compound at retention time 5.72 min in web extract of adult female *Latrodectus hesperus* (Fig. S1) analyzed by high performance liquid chromatography-mass spectrometry.


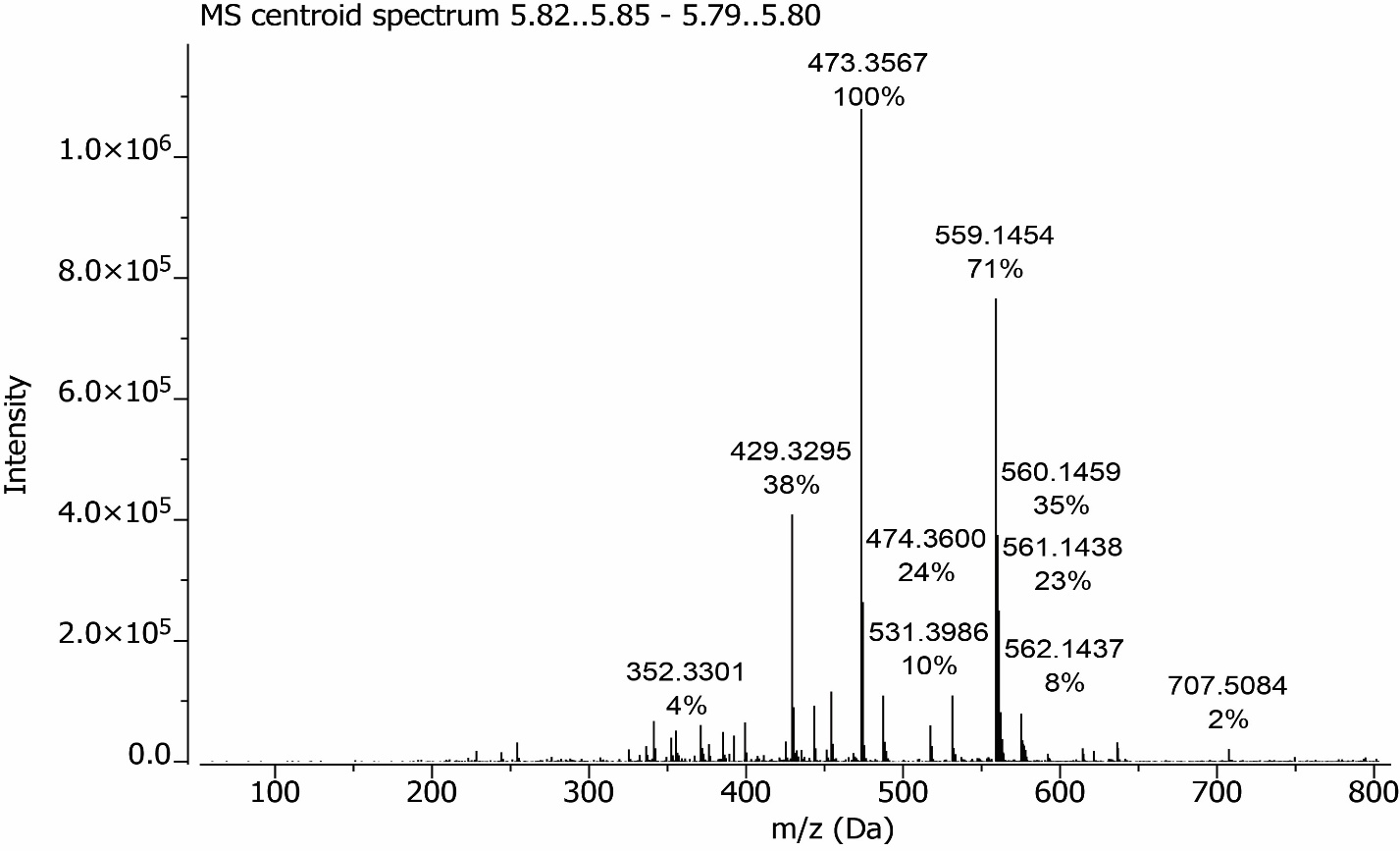


**Figure S12:** Positive electrospray ionization spectrum of the compound at retention time 5.83 min in web extract of adult female *Latrodectus hesperus* (Fig. S1) analyzed by high performance liquid chromatography-mass spectrometry.


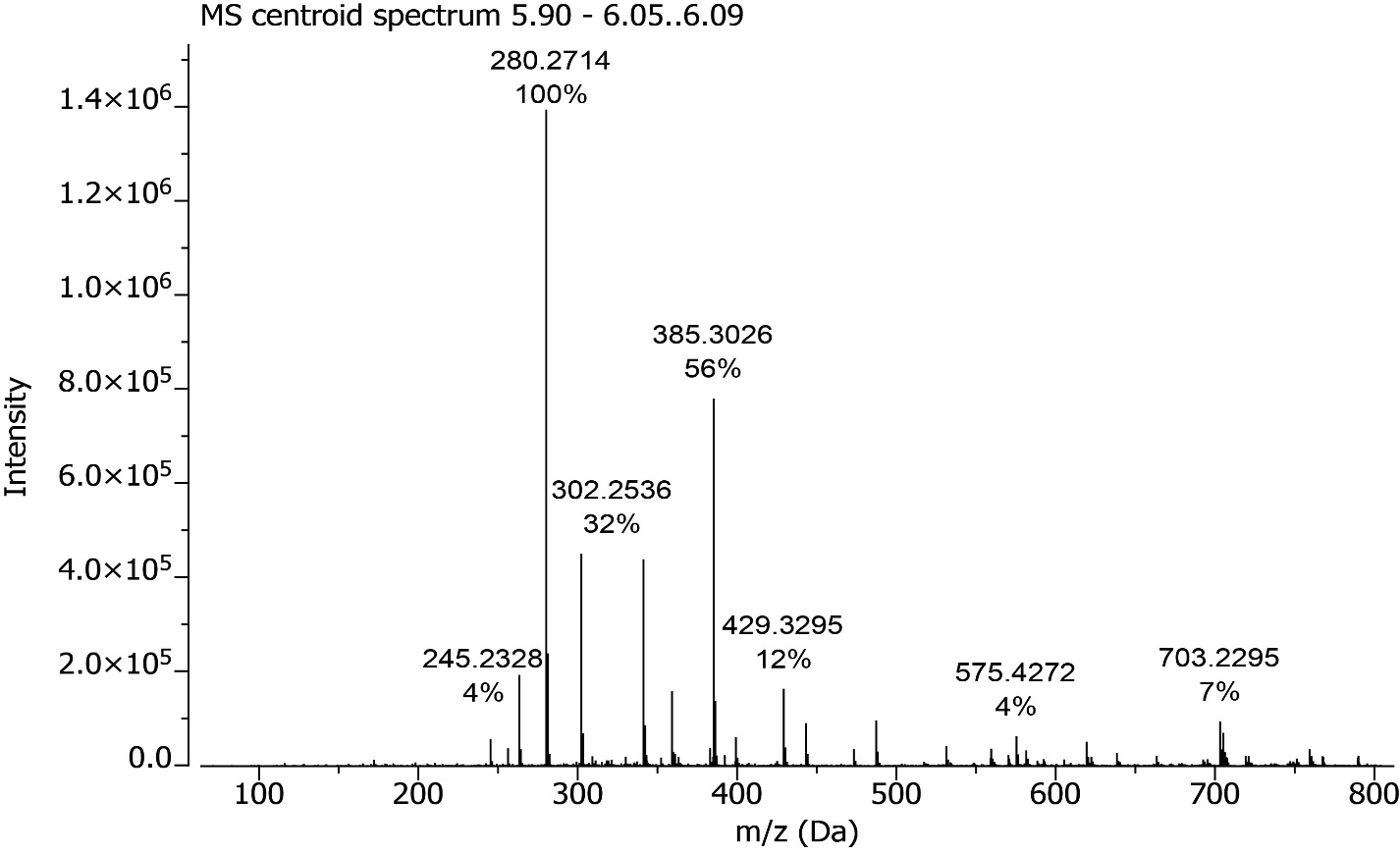


**Figure S13:** Positive electrospray ionization spectrum of the compound at retention time 5.95 min in web extract of adult female *Latrodectus hesperus* (Fig. S1) analyzed by high performance liquid chromatography-mass spectrometry.


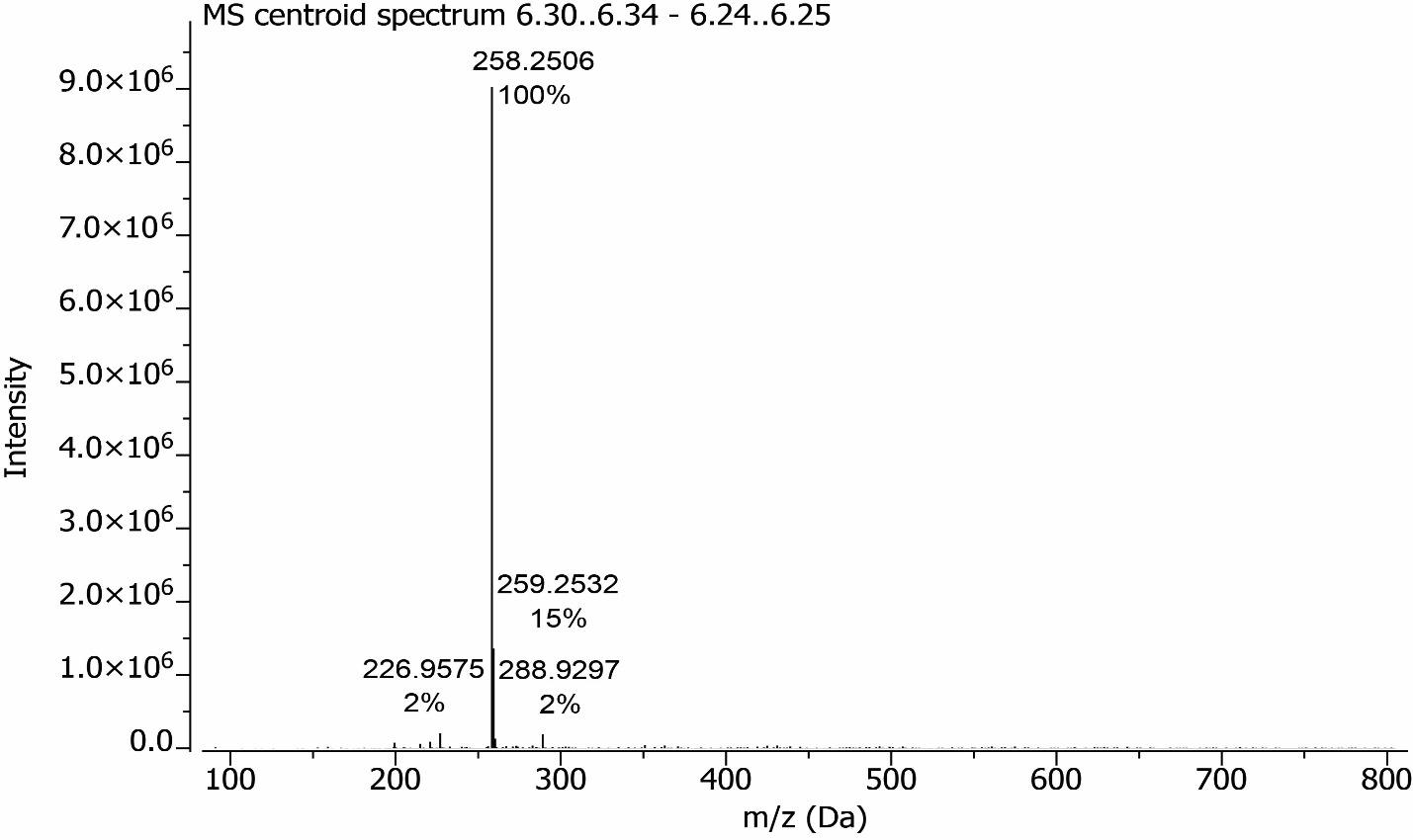


**Figure S14:** Positive electrospray ionization spectrum of the compound at retention time 6.32 min in web extract of adult female *Latrodectus hesperus* (Fig. S1) analyzed by high performance liquid chromatography-mass spectrometry.


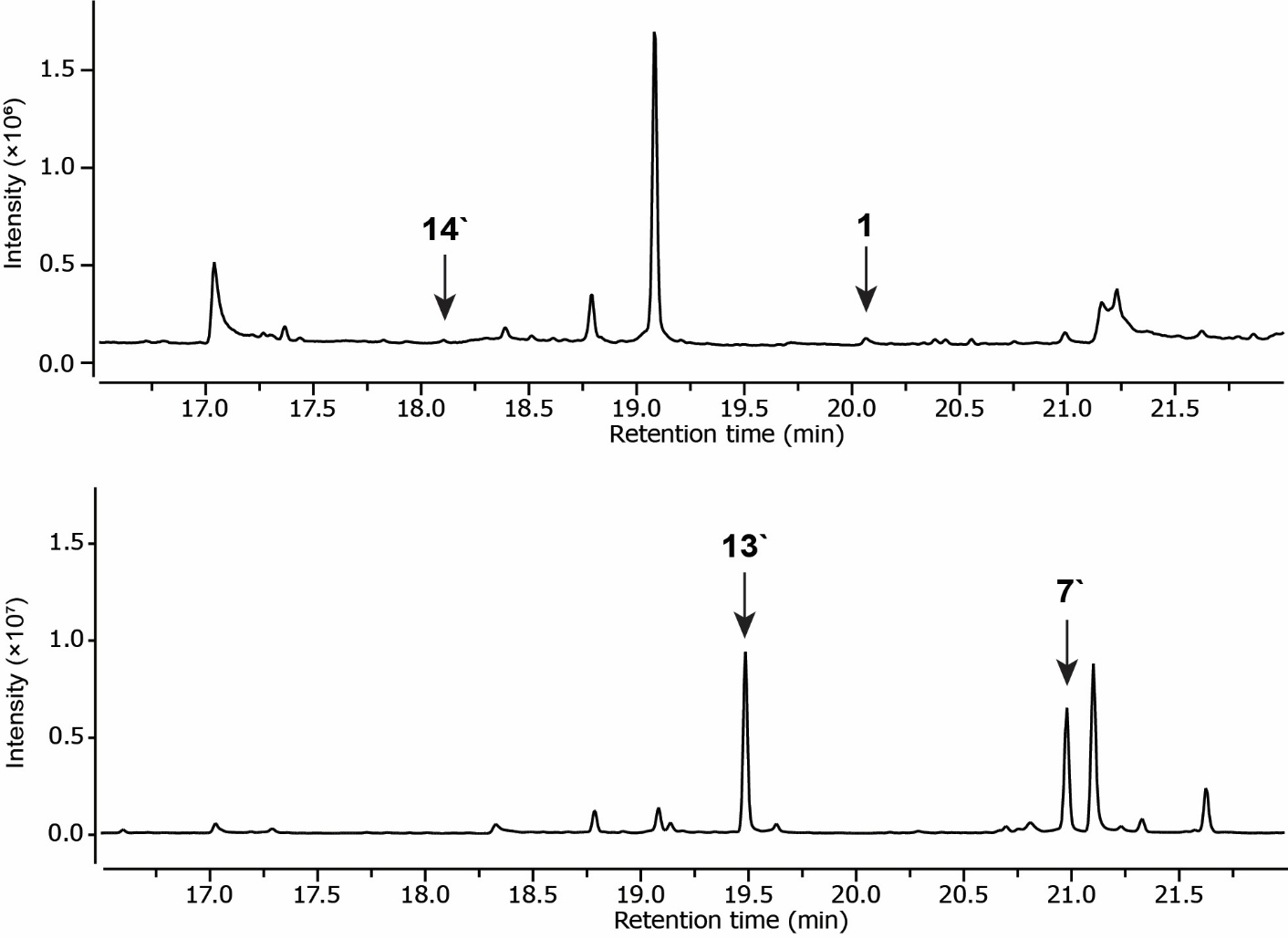


**Figure S15:** Total ion chromatograms (TICs) of web extracts of adult female *Latrodectus hesperus* derivatized by *N*,*O*-bistrimethylsilyltrifluoroacetamide (BSTFA) and analysed by gas chromatography-mass spectrometry. Both TICs in combination represent all compounds of interest: **1** (*N*-3-methylbutanoyl-*O*-isobutanoyl-L-serine methyl ester), **7** (*N*-3-methylbutanoyl-*O*-isobutanoyl-L-serine, **13** (*N*-3-methylbutanoyl-L-serine), and **14** (*N*-3-methylbutanoyl-L-serine methyl ester); note: compounds with apostrophe are derivatized.

**
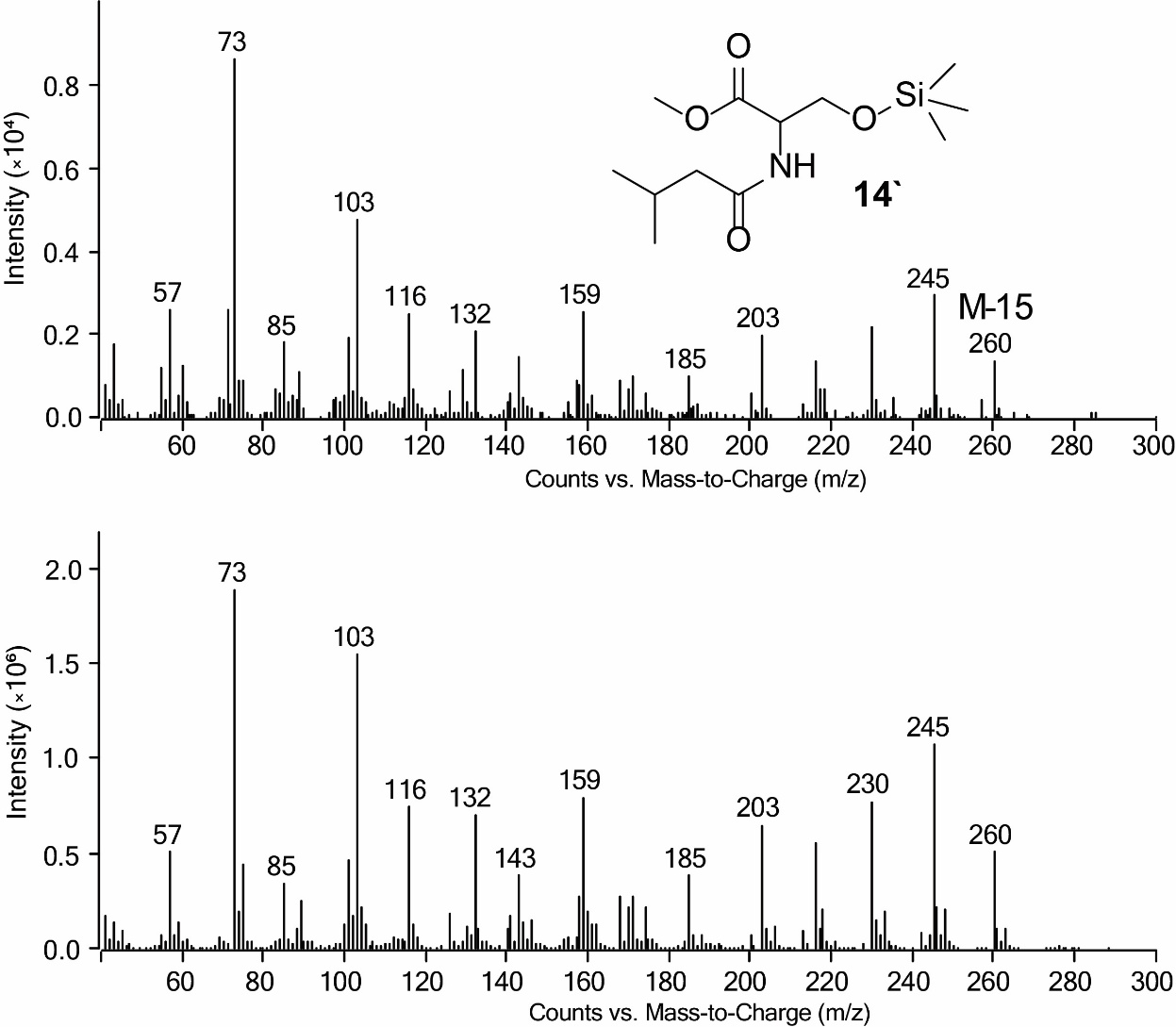
Figure S16:** Electron ionization spectra of BSTFA-derivatized (denoted by `) *N*-3-methylbutanoyl-L-serine methyl ester (**14**). The spectrum in the top subpanel represents derivatized **14`** in web extract of female *Latrodectus hesperus* (Fig. S15), whereas the spectrum in the lower subpanel represents synthetic derivatized **14**. BSTFA = *N*,*O*-bistrimethylsilyltrifluoroacetamide.


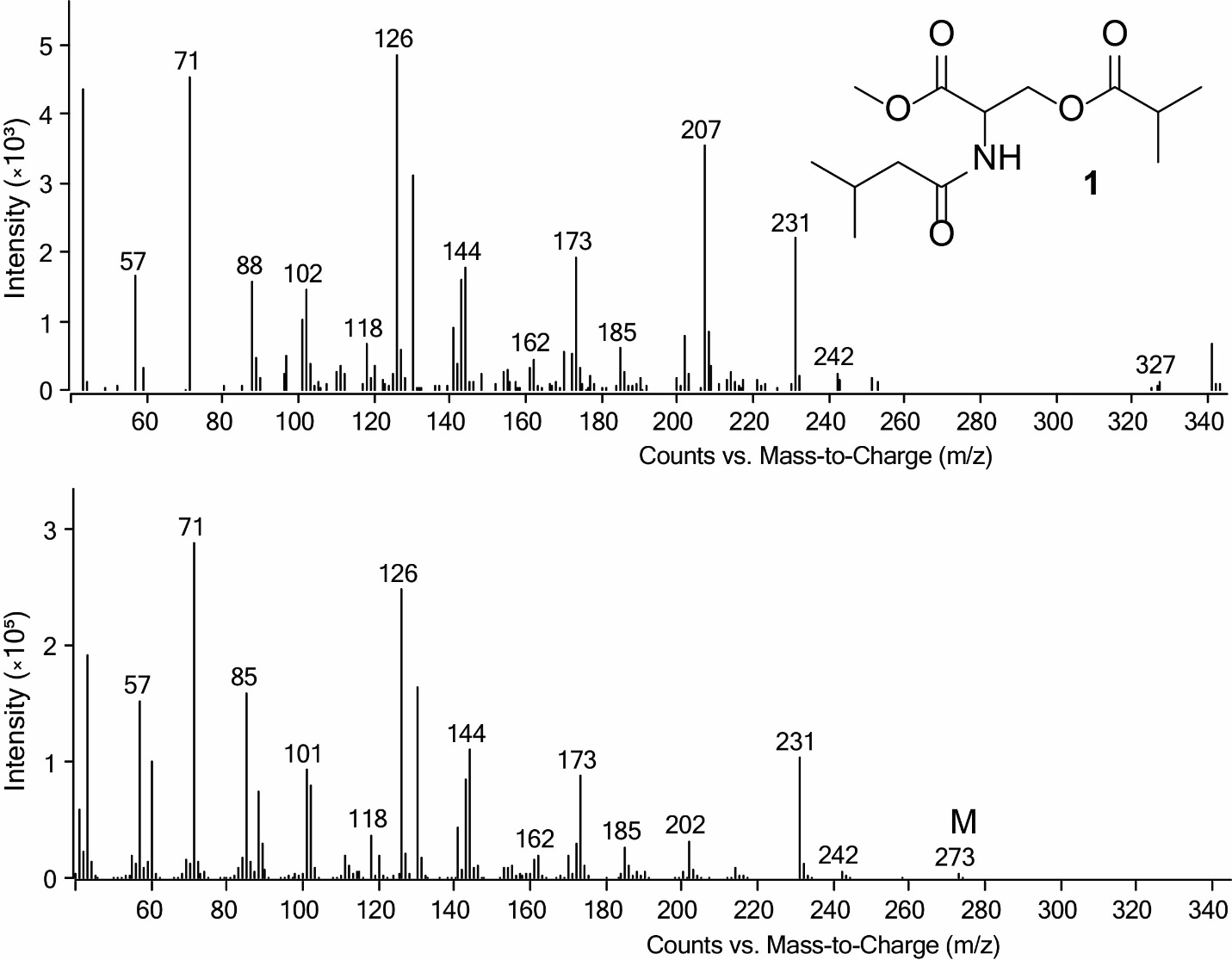


**Figure S17:** Electron ionization spectra of *N*-3-methylbutanoyl-*O*-isobutanoyl-L-serine methyl ester (**1**). The spectrum in the top subpanel represents **1** in web extract of female *Latrodectus hesperus* (Fig. S15), whereas the spectrum in the bottom subpanel represents synthetic **1**.


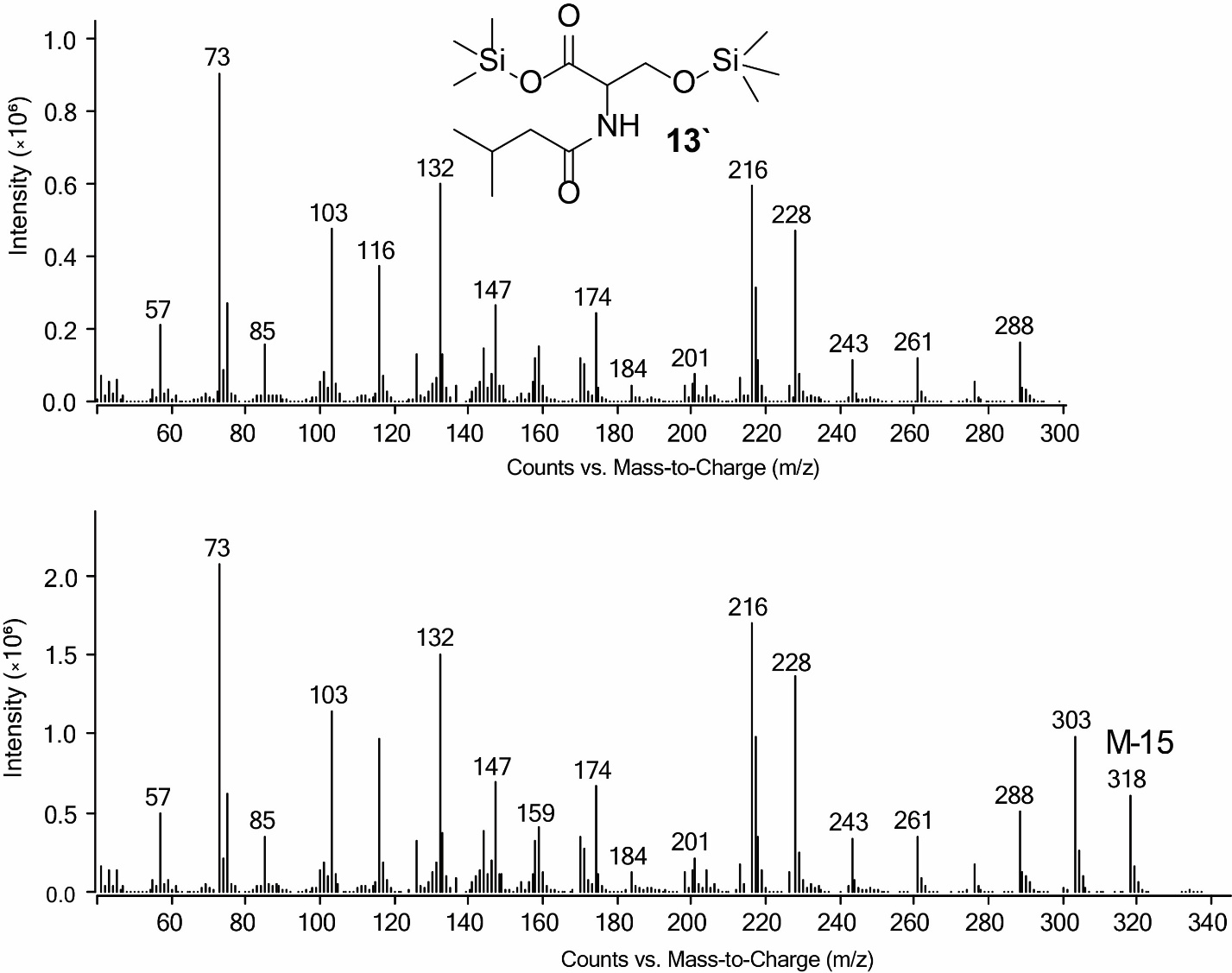

**Figure S18:** Electron ionization spectra of BSTFA-derivatized (denoted by `) *N*-3-methylbutanoyl-L-serine (**13**). The spectrum in the top subpanel represents derivatized **13`** in web extract of female *Latrodectus hesperus* (Fig. S15), whereas the spectrum in the bottom subpanel represents synthetic derivatized **13`**. Note: (1) BSTFA = *N*,*O*-bistrimethylsilyltrifluoroacetamide ; (2) due to a technical error, mass > 300 Da was not recorded for **13** in web extract.


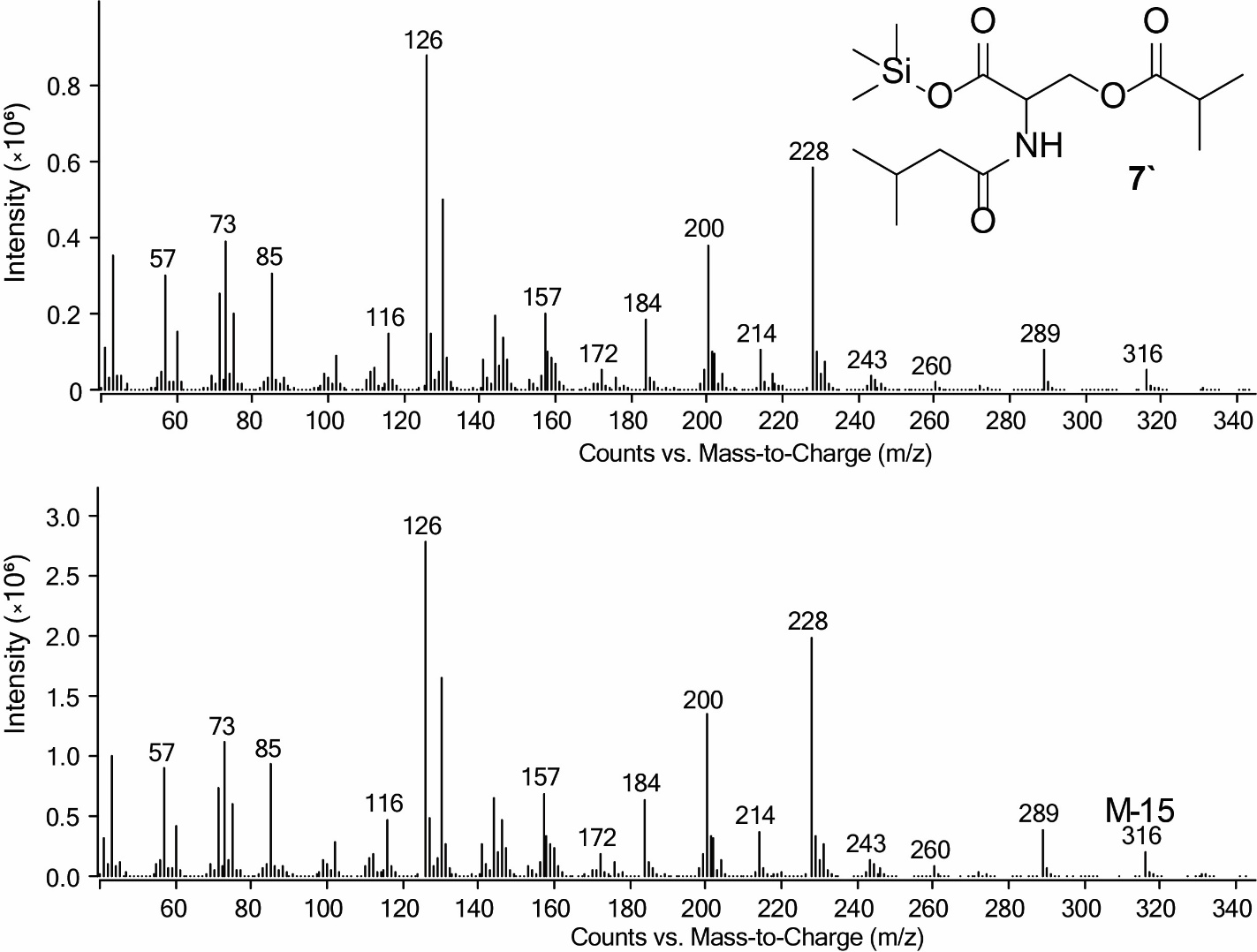
**Figure S19:** Electron ionization spectra of BSTFA-derivatized (denoted by `) *N*-3-methylbutanoyl-*O*-isobutanoyl-L-serine (**7**). The spectrum in the top subpanel represents derivatized **7`** in web extract of female *Latrodectus hesperus* (Fig. S15), whereas the spectrum in the bottom subpanel represents synthetic derivatized **7`**. BSTFA = *N*,*O*-bistrimethylsilyltrifluoroacetamide.
